# Utilisation of waste Cu-, Mn- and Fe-loaded zeolites generated after wastewater treatment as catalysts for air treatment

**DOI:** 10.3389/fchem.2022.1039716

**Published:** 2022-12-02

**Authors:** Mia Stanković, Margarita Popova, Matjaž Mazaj, Goran Dražić, Andraž Šuligoj, Nigel Van de Velde, Mojca Opresnik, Željko Jaćimović, Nataša Novak Tušar, Nataša Zabukovec Logar

**Affiliations:** ^1^ Faculty of Metallurgy and Technology, University of Montenegro, Podgorica, Montenegro; ^2^ Institute of Organic Chemistry with Centre of Phytochemistry, Bulgarian Academy of Sciences, Sofia, Bulgaria; ^3^ Department of Inorganic Chemistry and Technology, National Institute of Chemistry, Ljubljana, Slovenia; ^4^ Department of Materials Chemistry, National Institute of Chemistry, Ljubljana, Slovenia; ^5^ Faculty of Chemistry and Chemical Technology, University of Ljubljana, Ljubljana, Slovenia; ^6^ Graduate School, University of Nova Gorica, Nova Gorica, Slovenia

**Keywords:** clinoptilolite, zeolite 4A, metals removal, wastewater treatment, VOC removal, air treatment, waste-to-raw material

## Abstract

Disposal of copper, manganese and iron is particularly problematic in wastewater of metallurgical and galvanization plants, the electronics industry and agriculture. On the other hand, volatile organic compounds (VOCs), emitted from industrial processes, transportation and consumer products are the main class of air pollutants. The study revealed the potential of waste metal-loaded zeolite, generated through wastewater treatment procedures, to be utilised as an effective VOC removal catalyst for air treatment. In the first step, we have evaluated the sorption performance of natural zeolite clinoptilolite (HEU type), and synthetic zeolite 4A (LTA type) for the simultaneous removal of Cu^2+^, Mn^2+^ and Fe^3+^ species from aqueous solution. By a detailed sorption study, we determined the optimum sorption conditions and maximum metal concentrations in wastewater that can be after treatment disposed of in rivers or municipal plants. The efficiency of both zeolites for metal immobilization was demonstrated for concentrations up to 5 mg metals/1 g zeolite. These waste Cu-, Mn- and Fe-loaded zeolites were thermally treated at 540 °C before the second step, where we evaluated their catalytic performance in removing VOC. The thermally treated waste Cu-, Mn- and Fe-loaded natural zeolite clinoptilolite showed good catalytic performance in total toluene oxidation as a model VOC (conversion rate up to 96% at 510°C) and cycling stability (less than 15% drop in conversion rate in 4 h). In contrast, this is not the case for thermally treated waste Cu-, Mn- and Fe-loaded synthetic zeolite 4A.

## 1 Introduction

The contamination with heavy metals exists in the aqueous waste streams of many industries, including metallurgy, dyes and textile industry, mining operations, etc. (Vareda et al., 2019). Treatment processes for their removal from wastewaters include coagulation and chemical precipitation, membrane technologies, adsorption, etc. (Malik et al., 2019; Abdullah et al., 2019; Chen et al., 2018; Han et al., 2019; Siyal et al., 2018; Chmielewska, 2019a) Natural and synthetic zeolites have been recognized as low-cost, non-toxic and highly selective sorbents in heavy metal pollution control, especially in waste-water management. Comprehensive studies of metals’ sorption from water solutions on natural clinoptilolite tuffs have shown their great potential for the immobilization of some of the most toxic cations and anions of metals and metalloids like cadmium, chromium, lead and arsenic (Chmielewska, 2019a; Chmielewska, 2019b; Margeta et al., 2013; Yuna, 2016; Margeta et al., 2015). Iron, manganese and copper are very commonly found in large quantities in wastewaters of metallurgical and mining plants. Although at low concentrations they are considered less toxic, the European Parliament’s directive sets the limits for drinking water of these metals to copper 2 mg L^−1^, iron 0.2 mg L^−1^ and manganese 0.05 mg L^−1^ (Directive 2020/2184, 2020). Excess amounts of manganese and iron are also quite frequently found in some groundwater due to the presence of naturally occurring iron and manganese minerals, which are released into waters by weathering processes (Masindi and Muedi, 2018). In previous studies, we and other researchers showed that the natural zeolite clinoptilolite has excellent uptake capacity for manganese, copper and iron, as single type metals (Margeta et al., 2015; Rajic et al., 2009; Krstic et al., 2018; Stojaković et al., 2011; Kaplanec et al., 2017). Simultaneous removal of two metals was studied for Pb, Cd, Zn and Ni (Nuic et al., 2019; Stojakovic et al., 2016). Furthermore, the studies on the reversibility of the metal uptake revealed that it mostly depends on the type of metal due to the different mechanism of uptake (ion-exchange or absorption). For example, post-treatment of the samples with ammonium and sodium salts revealed irreversible sorption of manganese and iron and mostly reversible sorption of copper (Margeta et al., 2015).

On the other hand, with a thermal treatment of metal-loaded zeolites, also clinoptilolite tuffs, metal oxide particles are formed, i.e., after complete dehydration of zeolites above 500°C (Rajic et al., 2011), which suggests that they can be used as catalysts in oxidation reactions. Namely, the transition-metal-loaded synthetic zeolites and porous silicates are already considered promising low-cost catalysts for catalytic oxidation of volatile organic compounds (VOC) from industrial air as an alternative to noble-metal catalysts which are currently in use (Barakat et al., 2011; Djinovic et al., 2020). Toluene is often used as VOC probe molecule since it is one of the most common solvents in the chemical and processing industries, it is highly toxic, carcinogenic and also has an important POCP (Photochemical Ozone Creativity Potential) (He et al., 2019). Emissions to the atmosphere result from point sources (e.g., production) and area sources (e.g., marketing and use of petrol). Mean ambient air concentrations of toluene in rural areas are generally less than 5 mg m^−3^, while urban air concentrations are in the range 5–150 mg m^−3^ (WHO, 2000). Close to industrial emission sources concentrations may be higher. The Office of Environmental Health Hazard Assessment (OEHHA) is required to develop guidelines for conducting health risk assessments under the Air Toxics Hot Spots Program (Toluene reference exposure levels, Air Toxics Hot Spots Program, 2020). The guideline for toluene acute reference exposure levels is 5 mg m^−3^ and for toluene chronic reference exposer level is 0.42 mg m^−3^. The idea to use natural clinoptilolite as a support for stabilization of transition metals for their use in total toluene oxidation was already checked. In most of the studies the metal loading was optimized to obtain highly active metal-oxo-species on zeolite support. The tested systems were single metal oxides (Mn, Co., Fe, Cu) supported on clinoptilolite (Ozcelik et al., 2009; Soylu et al., 2010; Russo et al., 2018a; Russo et al., 2018b), bimetal oxides (Mn/Ni, Fe/Cu) supported on clinoptilolite (Ahmadi et al., 2017; Rostami and Jonidi Jafari, 2014) and single metals (Mn, Cu, Ni) supported on CeO_2_-clinoptilolite systems (Yosefi et al., 2015a; Yosefi et al., 2015b; Yosefi et al., 2015c). The catalysts prepared by incipient wetness impregnation of clinoptilolite in hydrogen form, which resulted in 9.5% MnO_2_ on clinoptilolite, exhibited the highest performance among different oxides in comparison with Fe, Cu and Co-oxides on the same support (Soylu et al., 2010). Its higher activity was attributed to its ability to maintain its redox potential. Furthermore, the catalyst appeared as active as conventional synthetic zeolites ZSM-5 and Beta. In another study (Ahmadi et al., 2017), the Mn and Ni were loaded on clinoptilolite by sonochemical method and the presence of single and mixed metal oxide nanoparticles was confirmed. Furthermore, the catalytic tests for total toluene oxidation revealed that bimetallic oxides on clinoptilolite support had lower temperature activity compared to monometallic ones and the Mn7%Ni3%/clinoptilolite sample showed the best performance. The toluene removal was achieved at 225°C and the removal efficiency remained constant for 1,440 min.

In our study, we focused on the performance of zeolites as adsorbents in close-to-real waste-water systems, by investigating in detail the simultaneous removal of three very common heavy metals and, in a second step, on the potential of waste zeolite as a raw material, i.e., as catalyst, in air treatment, more specifically for the removal of toluene as a model VOC. Namely, the excellent performance of some natural and synthetic zeolites in wastewater treatment, there are few studies that address the use of exhausted zeolites, when the metal-loaded zeolite cannot be regenerated for further use. In such cases, it is considered waste and usually disposed of in landfills or used in the cement industry.

## 2 Materials and methods

### 2.1 preparation of samples

The sorption experiments were performed by using natural zeolite sample from Brus deposit in Serbia and commercial zeolite 4A (Silkem Company, Slovenia). The natural zeolite samples were first crushed in an agate mortar and washed with distilled water in order to remove the surface dust. Grain size fractions up to 0.1 mm were chosen for the experiment. 10.00 g of each zeolite sample was pretreated with 500 ml of 2 mol L-1 solutions of NaCl to obtain Na-form zeolite (NaZ). The mixture was stirred for 48 h by a magnetic stirrer at 25°C. After the treatment, the samples were carefully washed with distilled water and dried in the oven at 60°C.

The Na-modified form of zeolite sample (NaZ) and commercially available zeolite 4A sample (4A) were placed in mixed solution of Cu(NO3)2.3H2O, Mn(NO3)2.4H2O and Fe(NO3)3.9H2O with different metal concentrations (from 0.5, 5 and 50 mg L-1 of each metal). 1.00 g of each sample was mixed with the 100 ml of solution for 0.5 h, 2 h, and 24 h at 25°C. The pH values before and after metal treatment were in the range of 5.5–6.0 for all samples for NaZ, while for 4A sample the pH before and after metal treatment was in the range of 7.5–9.0. Leaching of Na^+^ from the structure might be associated with both the Me2+ (Me = Cu, Mn or Fe) and H+ sorption, the latter causing the slight pH rise in the solution. At the end of the treatments the samples were separated by decantation and filtration to separate solutions for elemental analysis. Then, the samples were washed thoroughly with distilled water and dried at 60°C.

The as-prepared samples, denoted S-CuFeMn-NaZ-n and S-CuFeMn-4A-n (*n* = concentration of one metal type in mg L-1) were calcined at 540°C using heating rate of 10°C/min under air flow (20 ml/min) to obtain calc-CuFeMn-NaZ-n and calc-CuFeMn-4A-n. The samples after catalytic test (total toluene oxidation) were denoted postcalc-CuFeMn-NaZ-n and postcalc-CuFeMn-4A-n.

For comparative catalytic tests, single metal samples calc-Cu-NaZ-n, calc-Mn-NaZ-n and calc-Fe-NaZ-n (*n* = 50 and 150) were prepared following the same procedure as for mixed metal samples.

### 2.2 Characterization

The concentrations of iron, copper and manganese in solutions were determined with Inductively-Coupled Plasma Atomic Emission Spectrometry (ICP-AES) on Thermo Jarrell Ash, model Atomscan 25. The accuracy of the measurements is ±5% for the lowest concentrations.

The X-ray powder diffraction data of the samples were collected on a PANalytical X’Pert PRO high-resolution diffractometer with CuKα1 radiation (λ = 1.5406 Å) in the range from 5 to 80° 2θ with the step of 0.034° per 100 s using fully opened 100 channel X’Celerator detector. The qualitative powder analyses of the collected XRD patterns were performed using the HighScore Plus Software Package (HighScore Plus Software Package 4.9, 2020).

Morphological properties of the samples were observed by scanning electron microscopy measurements (SEM) on Zeiss Supra™ 3VP field-emission gun (FEG) microscope. Elemental analysis was performed by energy dispersive X-ray analysis (EDAX) with an INCA Energy system attached to the above-described microscope and by Perkin Elmer 2,400 Series II CHNS analyser in bulk sample, coated with carbon. An average elemental composition of the sample using EDXS was obtained by a data collection at five different mm2-sized windows on the sample surface. The accuracy of the measured element contributions is ± 3%.

FTIR spectroscopy using attenuated total reflectance (ATR) apparatus were carried out by using an Alpha–Bruker FTIR spectrometer using a diamond crystal in a horizontal position. ATR-FTIR spectra were collected in the spectral region from 4,000 to 400 cm^−1^ with a resolution of 4 cm^−1^.

Diffuse reflectance absorption spectra of the materials were recorded on a Lambda 650 (Perkin Elmer) UV–vis spectrophotometer, equipped with a Praying Mantis accessory (Harrick). The scan speed was 480 nm/min and the slit was set to 2 nm. Spectralon^®^ was used for background correction.

The Raman spectra were recorded in the spectral range from 70 to 3,600 cm^−1^ using an Alpha 300 RA confocal microscope (Witec, Ulm, Germany) with a green laser excitation at 532 nm, and accumulation time of 40 or 50 s with a resolution of 4 cm^−1^. For each sample, at least three different locations were analysed.

High-resolution transmission electron microscopy (HR-TEM) on postcalc-CuFeMn–NaZ-50 sample was performed on a 200-kV field-emission gun (FEG) microscope JEOL JEM 2100. Samples were dispersed in ethanol and placed on a copper holey carbon grid. The specimens were additionally coated with carbon in order to prevent excessive charging of the samples under the electron beam. ADF-STEM and BF-STEM images and EELS spectra on calc-CuFeMn–NaZ-50 and calc-CuFeMn-4A samples were obtained by probe Cs corrected Scanning TEM Jeol ARM 200 CF operated at 80 kV to minimize electron beam damage equipped with a Gatan Quantum ER Dual EELS system.

The thermal analysis (TG/DTG) was performed on a Q5000 IR thermogravimeter (TA Instruments, Inc.). The measurements were carried out in air flow (10 ml/min) with the heating rate of 10°C/min.

The specific surface areas were determined by BET method based on the N2 and Ar sorption isotherms measured at 77 K and 87 K respectively by using Quantachrome iQ3 sorption analyser. CO2 isotherms were performed on HTP-IMI manometric sorption analyser (Hiden Isochema Inc.) at 273 K. Samples were degassed at 200°C for 16 h prior the measurements were carried out.

### 2.3 Catalytic test reaction procedure

Toluene oxidation was studied at atmospheric pressure using a fixed-bed flow reactor, air as carrier gas and 200 mg sample. The air stream passed through a saturator filled with toluene and equilibrated at 0°C (ptoluene = 0.9 kPa, 1%). The activity was determined in the temperature interval of 300–510°C at WHSV (weight hourly space velocity) of 1.2 h^−1^. The reactor itself was a quartz tube of 15 mm inner diameter, with the catalyst bed in the middle. A thermocouple was positioned in the catalyst bed for accurate measurement of the catalyst temperature. All gas lines of the apparatus were heated to 110°C in order to minimize VOC adsorption on the tube walls. The on-line gas chromatographic analysis was performed on NEXIS GC-2030 ATF with PLOT Q column using a thermal conductivity detector. Toluene conversion was calculated as Xtol = (C°tol - Ctol)/C°tol × 100 where C°tol and Ctol are the moles of toluene in the inlet flow and moles of non-converted toluene. CO2 and water were the only products, which were detected during the catalytic reaction. The products of partial oxidation (benzaldehyde and benzoic acid) were not detected. The determined carbon mass balance is 98–100% in all catalytic experiments.

## 3 Results and discussion

### 3.1 Study of metal loading

The affinity of both zeolites to immobilize selected metals was evaluated by the elemental analysis of metal solutions after treatment. Based on our previous studies, the Na-exchanged form of zeolite tuff was used in sorption study, because the treatment improved the uptake of all investigated metal cations in comparison with the unmodified zeolites (Margeta et al., 2015). This is mostly because the ion-exchange process is more controlled when only one type of extra-framework cations is present in zeolite, instead of four or more (e.g. Na^+^, Ca^2+^, K^+^ and Mg^2+^ in clinoptilolite). Synthetic zeolite 4A is produced in Na-form only. We choose metal salts, where metals are in oxidation states Fe^3+^, Mn^2+^ and Cu^2+^. These are the most common forms of selected metals in water at neutral and slightly acidic conditions. For the adsorption kinetic studies we focused only on two limit cases i.e., 30 min and 24 h.

The concentrations of the metals in solutions before and after sorption on zeolites are presented in Table 1.

**TABLE 1:** Concentrations of the three metals in the final solutions after sorption treatment after selected time of the experiment and concentrations of the three metals in the starting solutions.

Sample	Concentration of each metal in starting solution (mg L^−1^)	Time (h)	Fe (mg L^−1^)	Cu (mg L^−1^)	Mn (mg L^−1^)
S-CuFeMn–NaZ-50	50	0.5	15	20	17
S-CuFeMn–NaZ-5	5	0.5	1.6	2.4	2.6
S-CuFeMn–NaZ-0.5	0.5	0.5	< 0.1	< 0.1	< 0.1
S-CuFeMn–NaZ-50	50	24	11	14	15
S-CuFeMn–NaZ-5	5	24	0.3	0.5	0.9
S-CuFeMn–NaZ-0.5	0.5	24	< 0.1	< 0.1	< 0.1
S-CuFeMn–4A-50	50	0.5	4	8	10
S-CuFeMn–4A-5	5	0.5	< 0.1	< 0.1	0.6
S-CuFeMn–4A-0.5	0.5	0.5	< 0.1	< 0.1	< 0.1
S-CuFeMn–4A-50	50	24	7	8	11
S-CuFeMn–4A-5	5	24	0.6	0.4	0.5
S-CuFeMn–4A-0.5	0.5	24	0.4	0.1	0.1

Both zeolites showed high sorption capacities and rates for metal immobilization in a wide metal (Me) concentration range. At the lowest starting concentration (0.5 mg of each Me/g of zeolite; Me = copper, iron and manganese in the aqueous solutions) both sorbents were capable of removing more than 99% of metal cations from the solutions already after 30 min of exchange process.

With ten times higher metal concentration (5 mg L^−1^) the level of their removal remains very high. It is evident that the sorption kinetic rates are significantly inhibited if compared to the most diluted system. Nevertheless, the amount of metal cations within aqueous solutions drops to 0.4 mg L^−1^, 0.5 mg L^−1^ and 0.3 mg L^−1^ for Mn^2+^, Cu^2+^ and Fe^3+^ respectively after 24 h of exchange. The concentrations of all three metals are therefore below the threshold, i.e., the maximal concentrations in wastewaters that can be disposed of in a river or municipal plant (Directive 2020/2184, 2020).

In the highest metal concentration systems (50 mg L^−1^) the zeolites are capable to remove up to 78% of Fe^3+^, 72% of Cu^2+^ and 70% of Mn^2+^ after 24 h of treatment. With synthetic zeolite 4A we can remove up to 92% of Fe^3+^, up to 84% of Cu^2+^ and up to 80% of Mn^2+^ after 0.5 h of treatment. After 24 h of treatment, small amount of metals is desorbed from synthetic zeolite. In Supplementary Table S1 0.1 some literature data on sorption performance of natural and synthetic zeolites is summarized, which reveals comparable uptakes [e.g. Logar et al., 2021; Mokrzycki et al., 2022].

From obtained results in our study we deduced the selectivity of metal sorption to be in the following order Fe^3+^ < Cu^2+^ < Mn^2+^ and that both zeolite systems are able to efficiently remove the selected pollutant cations in a wide concentration range with zeolite 4A possessing slightly higher sorption capacities than clinoptilolite. This is in accordance with the higher ion-exchange capacity of zeolite 4A in comparison to clinoptilolite for Me^2+^ ions. The theoretical ion-exchange capacity for HEU-type zeolite sample with 71.1% of HEU-type zeolite (K_T_ (Me)) is 56,2 mg Me^2+^/g zeolite tuff and 223.6 mg Me^2+^/g zeolite for LTA-type zeolite (Cerjan Stefanovic et al., 2007). At the same time, we can conclude, that one metal cation does not completely exclude the immobilization of the other two metals, as it was found in the case of simultaneous sorption of Pb/Zn and Cd/Zn (Nuic et al., 2019).

### 3.2 Structure characterization

The results of the bulk EDX elemental analysis of metal-exchanged clinoptilolite tuff and zeolite A samples are presented in Table 2. After sorption of metals on both zeolites, the increased amount of manganese, iron and copper was associated by the reduced amount of sodium in NaZ and in 4A samples. The discrepancies between the amount of metal that remained in the solution after metal sorption and the amount of metal immobilized on zeolite are due to inhomogeneous distribution of metals and/or a partial leaching of the loaded metal cations from the zeolites during the thorough washing.

**TABLE 2:** An average elemental composition of selected samples obtained by the EDX analysis (in at%).

Sample	O	Na	Mg	Al	Si	K	Ca	Mn	Fe	Cu
NaZ	67.30	3.31	0.35	4.70	23.73	0.43	0.13	−	0.05	−
S-CuFeMn–NaZ-50 (0.5 h)	67.47	2.57	0.34	4.62	23.66	0.38	0.12	0.11	0.56	0.17
S-CuFeMn–NaZ-50 (24 h)	67.18	2.48	0.32	4.61	23.74	0.39	0.13	0.15	0.60	0.15
4A	62.88	11.96	−	12.56	12.60	−	−	−	−	−
S-CuFeMn–4A-50 (0.5 h)	63.23	11.03	−	12.46	12.68	−	−	0.17	0.21	0.21
S-CuFeMn–4A-50 (24 h)	62.51	10.84	−	12.79	13.23	−	−	0.19	0.24	0.21

SEM analysis of S-CuFeMn–NaZ-50 (24 h) and S-CuFeMn–4A-50 (24 h) samples (Supplementary Figure S4) reveals that natural zeolite clinoptiolite tuff exhibits characteristic clinoptilolite plate-like crystals with sizes up to 0.5 μm and zeolite 4A cubic crystals with sizes around 2 μm. Based on the TG analysis (Supplementary Figure S1 and literature data), both zeolites are thermally stable to at least 750°C. The calcination temperature was chosen to be 540°C, which is commonly used in calcination of zeolites, as it results in complete dehydration of the zeolite (Stojakovic et al., 2011).

For further structure investigations, calcination and catalytic tests S-CuFeMn–NaZ-50 (24 h) and S-CuFeMn–4A-50 (24 h) samples were selected (marked bold in Table 1 and Table 2).

**FIGURE 1:**
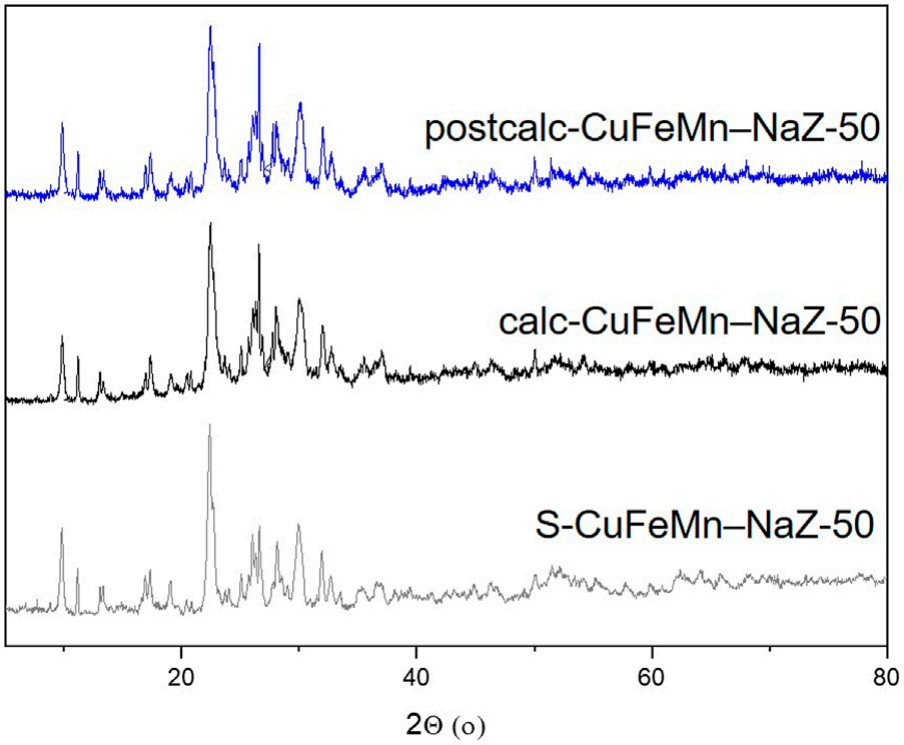
X-ray powder diffractograms of metal-modified S-CuFeMn–NaZ-50 (gray), calcined metal-loaded sample calc-CuFeMn–NaZ-50 (black) and calcined metal-loaded sample after catalytic test postcalc-CuFeMn–NaZ-50 (blue).

**FIGURE 2:**
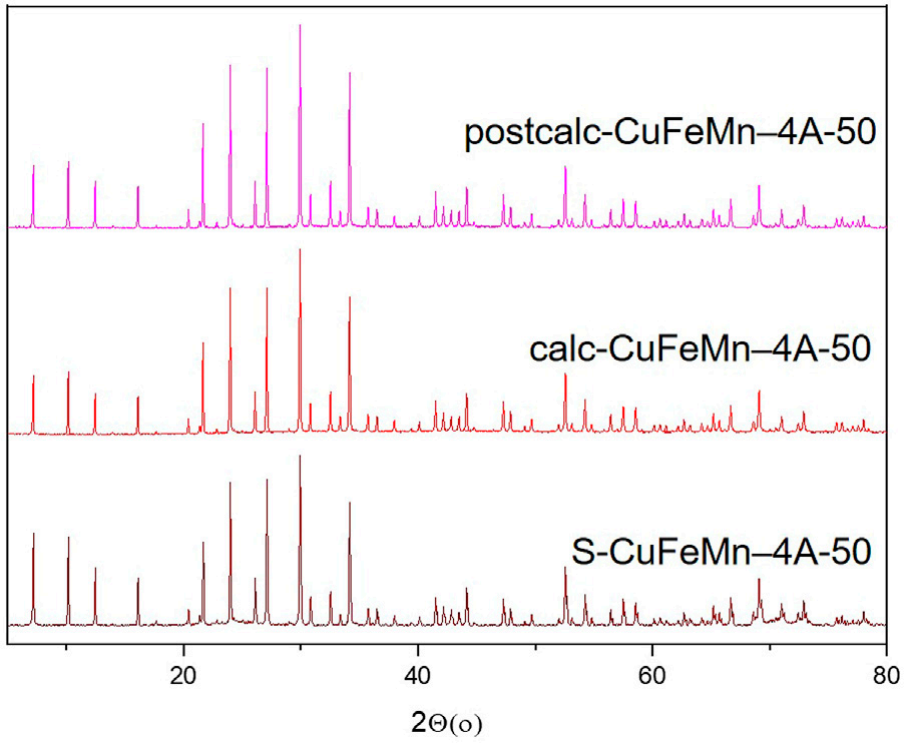
X-ray powder diffractograms of zeolite 4A, metal-modified S-CuFeMn–4A-50 (brown), calcined metal-loaded sample calc-CuFeMn–4A-50 (red) and calcined metal-loaded sample after catalytic test postcalc-CuFeMn–4A-50 (magenta).

The X-ray powder diffraction (XRD) patterns of NaZ, metal-modified S-CuFeMn–NaZ-50, calcined metal-loaded sample calc-CuFeMn–NaZ-50 and calcined metal-loaded sample after catalytic test postcalc-CuFeMn–NaZ-50 are shown in Figure 1. Quantitative XRD analysis, performed on a natural zeolite that we used in this study, showed that it consists of 76.2% of clinoptilolite, 11.3% of feldspar, 6.9% of quartz and 5.9% of mica (Margeta et al., 2015) (Supplementary Figure S5). On the other hand, XRD of the used synthetic zeolite confirms the presence of well crystalline zeolite 4A phase (98%) and sodalite phase impurities (2%), as shown in Figure 2 and Supplementary Figure S5. This is also valid for metal-modified S-CuFeMn–4A-50, calcined metal-loaded sample calc-CuFeMn–4A-50 and calcined metal-loaded sample after catalytic test postcalc-CuFeMn–4A-50.

The crystallinity of both types of zeolites (clinoptilolite and zeolite 4A) is not significantly reduced after the metal sorption and calcination, as revealed by XRD analysis. More importantly, it stays the same after the catalytic reaction. We have checked the XRD patterns for a possible presence of (mixed)metal oxides, however, with the present XRD data we could not confirm the formation of any metal-oxo/hydroxo species on the surface of the zeolites, which was reported in the literature (Ozcelik et al., 2009; Ahmadi et al., 2017). Based on that, we did not exclude the presence of metal-oxo-species, but we anticipated that they are not detectable by XRD due to their size, especially for the very complex (multiphase) natural zeolite samples. The XRD patterns of single metal loaded and calcined samples before and after catalytic test reactions are shown in Supplementary Figure S2.

**FIGURE 3:**
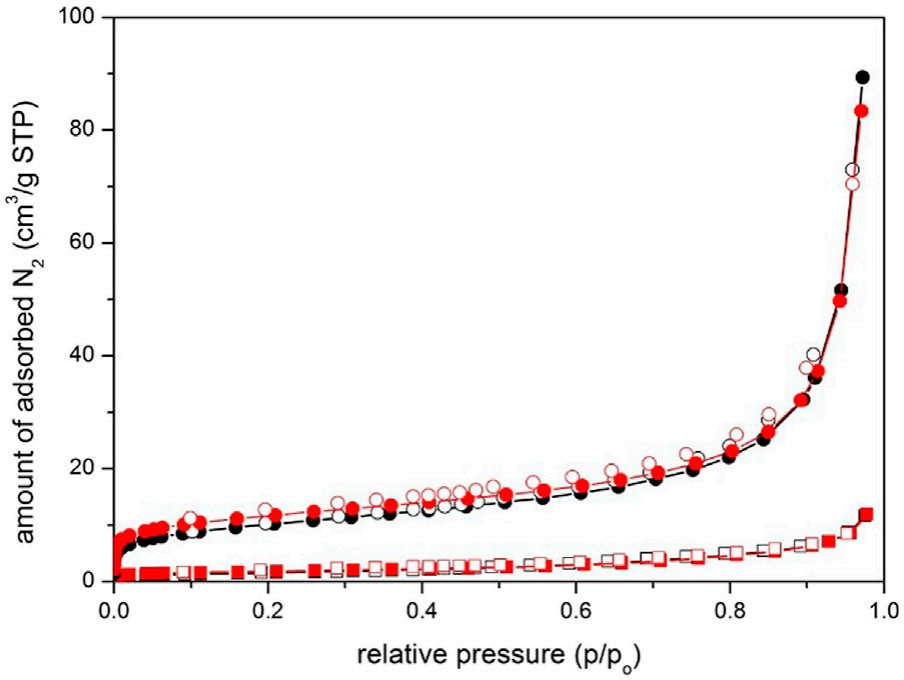
N_2_ sorption isotherms of S-CuFeMn-4A-50/calc-CuFeMn–4A-50 (squares) and S-CuFeMn-NaZ-50/calc-CuFeMn–NaZ-50 (circles) samples. Black plots show as-synthesized materials whereas red plots calcined (post-) materials. Full symbols—adsorption pointes, empty symbols - desorption points.

The N_2_ sorption isotherms analyses of S-CuFeMn–NaZ-50 and calc-CuFeMn–NaZ-50 as well as S-CuFeMn–4A-50 and calc-CuFeMn–4A-50 are shown on Figure 3 whereas corresponding BET values are given in Table 3.

**TABLE 3:** Specific surface area values of the investigated samples.

Sample	^1^SBET (m^2^ g^−1^)
S-CuFeMn-4A	5.7
calc-CuFeMn-4A	6.3
S-CuFeMn-NaZ	30.7
calc-CuFeMN-NaZ	37.3

BET, surface area values were calculated from p/p_o_ region selected from Roquerol plots.

Both zeolite systems possess low BET surface areas. The BET values for zeolite tuff are comparable with the ones reported before for the used zeolite, i.e., 35 m^2^/g, 22 m^2^/g, and 20 m^2^/g for unmodified clinoptilolite tuff, NaZ form and CaZ form (Cerjan Stefanovic et al., 2007). The BET surface areas for zeolite A samples are significantly lower. We may attribute this to the inaccessibility of the test adsorbate (N_2_) due to small pores entrances of both zeolites: zeolite 4A (0.41 nm in diameter), clinoptilolite (0.53 nm × 0.31 nm), while dynamic diameter of N_2_ molecule is 0.36 nm (Sotomayor et al., 2018; Villarroel-Rocha et al., 2020). The limited diffusion of N_2_ molecules into the pores of zeolite 4A can also be attributed to the location of Na^+^ cations at the entrance to the *α*-cages (Starke et al., 2021). However, to get a more detailed insight to the porosity properties, Ar and CO_2_ isotherms were also measured for zeolite 4A samples (Supplementary Figure S9, Supplementary Table S9). While argon adsorption measured at 87 K exhibit similar behaviour as nitrogen, the dimensions and polarizability of CO_2_ on the other hand allows the diffusion through narrow openings to access sodalite cage of zeolite 4A framework. Microporosity of zeolite 4A samples is thus revealed, when CO_2_ is used as a probe adsorbate, possessing the BET surface areas of 381 m^2^/g and 411 m^2^/g for S-CuFeMn–4A-50 and calc-CuFeMn–4A-50 samples, respectively. In the case of clinoptilolite-based samples, the contributions to the BET surface areas are mostly dependent on the particle shape, size and degree of their agglomeration. Clinoptilolite samples occurring in nanosized sheet-like crystallites possess significantly higher outer surface area than cubic-like micron-sized zeolite 4A. Additionally, aggregated clinoptilolite crystallites result in the occurrence of pronounced interparticle mesoporosity. The increase in BET after calcination at 550°C in both zeolite could be due to the two possible reasons: 1) heating at sufficiently high temperature can remove water molecules which can be strongly bonded to the extra-framework cation sites, partially occupying zeolitic micropores or interparticle voids; 2) deposited metal species are degraded to corresponding metal-oxide particles upon heating which remain to be located on the surface of zeolitic crystals, importantly contributing to the surface roughness and consequently increment of BET surface area.

**FIGURE 4:**
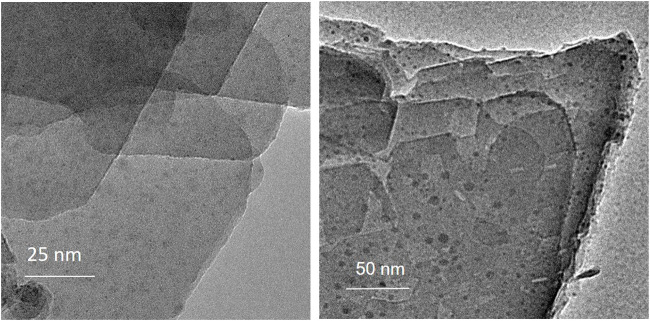
TEM images of calc-CuFeMn–NaZ-50 (left) and postcalc-CuFeMn–NaZ-50 (right).

TEM analysis of calc-CuFeMn–NaZ-50 and postcalc-CuFeMn–NaZ-50 samples confirmed the presence of metal oxide nanoparticles in both samples, evenly dispersed on the surface of the crystallites. The appearance of such nanoparticles originates from the calcination process of the zeolite being loaded with metal cations. Furthermore, the analysis showed that the size of metal-oxide particles did not change/agglomerate after the catalytic reaction, i.e. the average size was in both cases estimated to around 5 nm (Figure 4, Supplementary Figure S7).

TEM examination of calc-CuFeMn–4A-50 confirmed the presence of 1 μm zeolite 4A particles of cubic shape (Supplementary Figure S7). Further investigation for the presence of metal oxide particles was not possible due to the thickness of the crystals.

**FIGURE 5:**
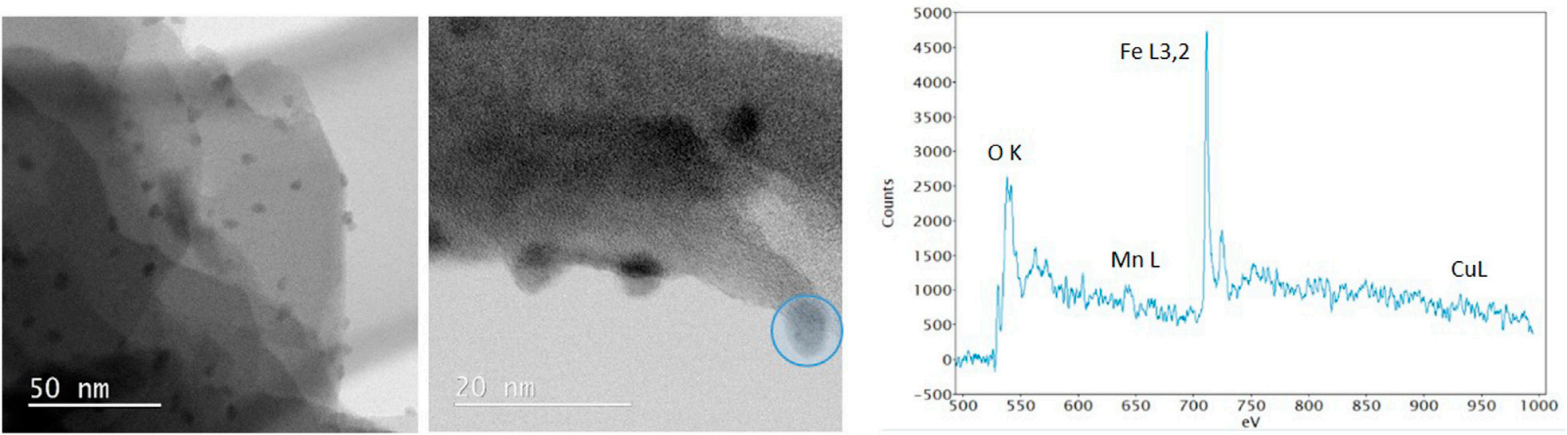
BF-STEM images with EELS spectrum of nanoparticle marked in blue circle of calc-CuFeMn–NaZ-50.

The BF-STEM images and associated EELS spectrum of a single nanoparticle in calc-CuFeMn–NaZ-50 (Figure 5) confirmed the presence of metal oxide phase. According to EDX analysis the Cu:Fe:Mn ratio is 1:4:1 (Table 2). However, the EELS spectrum revealed higher amount of Fe in relation to Mn and Cu, i.e., estimated ratios of Cu:Fe:Mn are <1%:94%:6%. We can assign this difference to the easier leaching of iron from the pores of zeolite to the external surface of the sample forming the detected oxide particles and/or the formation of additional thin surface Cu- and Mn- oxo phase, which could not be detected. Based on Fe L3/L2 intensity ratio we can assume that the iron is in 3 + valence state forming hematite-like phase.

For additional insight into the structure of metal-oxo species, we performed FTIR and Raman measurements. No changes were seen in ATR FTIR spectra amongst samples in the same group of materials (single-metal and mixed-metal loaded NaZ and 4A, Supplementary Figure S8).

**FIGURE 6:**
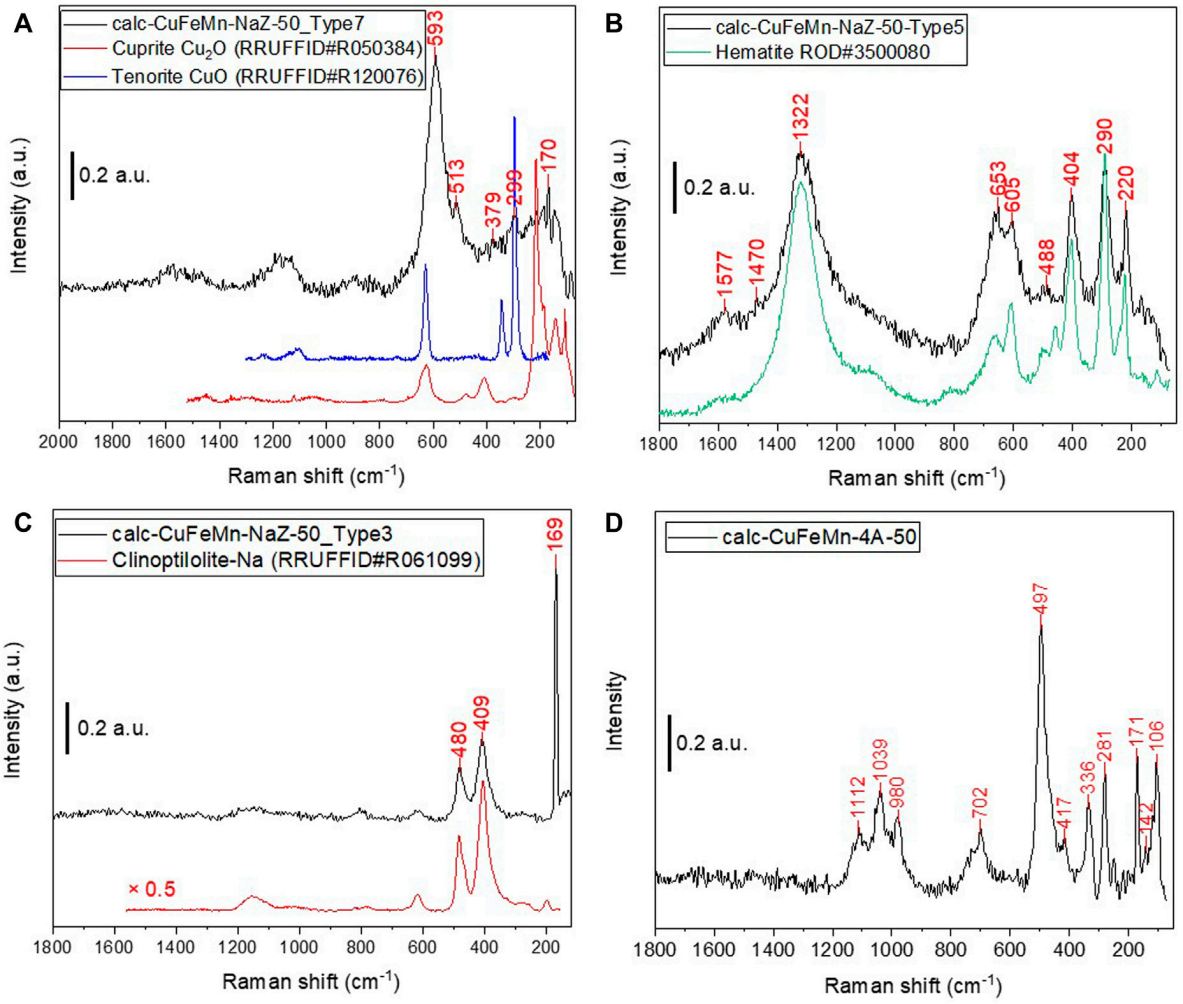
Raman spectra of sample calc-CuFeMnNaZ-150 **(A,B** and **C)** and sample calc-CuFeMn-4A-50 **(D)**. Detected peaks are labelled in red while appropriate references are put below spectra in A, B and C. Spectra are vertically translated for clarity.

Raman spectroscopy showed (Figure 6) that in sample calc-CuFeMn-NaZ-50 seven distinct Raman spectra regions can be seen, showing great heterogeneity of the sample. For example, region 7 (1 measurement position, Figure 6A) showed distinct peaks at 299 and 593 cm^−1^, possibly showing on the presence of CuO oxides (Deng et al., 2016). The peaks are slightly red shifted if compared to cuprite (Cu_2_O) or Tenorite (CuO), suggesting on the presence of mixed copper oxide. The region 5 (Figure 6B), which is the most commonly found region in the sample (4 measurement positions out of 11), shows prominent peaks at 1,322, 652, 605, 403, 290, and 220 cm^−1^. These correspond well to Fe_2_O_3_ hematite phase (ROD#3500080). Another type of spectra was also found (Type 1), which has a good match with hematite only red-shifted by 10–35 cm^−1^. The shift to lower frequencies is a potential indication on the presence of mixed oxides (Du et al., 2019). Although the shift can stem from the impact of the high-density laser beam, the fact that the shift was present in copper oxide and also in Fe_2_O_3_ phase supports the idea of the presence of mixed-metal oxides. Couple of other spots (2 measurement positions out of 11, Figure 6C) show absence of metal oxide nanoparticles and only hint on the presence of zeolitic phase (clinoptilolite). The presence of Mn species is hinted with Raman peaks at 634 and 508 cm^−1^, which were detected in one of the studied spots (Supplementary Figure S8). They could be attributed to the stretching mode of the MnO_6_ octahedra. (Julien et al., 2002; Post et al., 2021).

On the other hand, sample calc-CuFeMn-4A-50 (Figure 6D) shows much more homogeneous Raman spectra across the tested areas. No traces of metal oxides could be found in this sample. The vibrations were found at 281, 336, 417, and 497 cm^−1^, which are typically found in zeolite A (LTA topology). Most significant is the peak at 497 cm^−1^, corresponding to double 4-member rings symmetric breathing modes (combining 4-four to six and 4-four to eight tricyclic bridges) (Depla et al., 2011; Luo et al., 2021). These findings therefore exclude the possibility of the formation of large metal oxide particles in the zeolite A samples. Possible explanation for this result would be that the migration of metal cations loaded in the pores of zeolite A out of the pores to form metal oxides on the surface of zeolite is limited due to smaller pore openings and stronger interaction of metal cations to more electronegative zeolite A framework, if compared to clinoptilolite sample. For example, stronger interaction of Cu^2+^ with zeolite A framework than with clinoptilolite framework in Cu-modified zeolites was shown by X-ray absorption analysis (Logar et al., 2021). However, the formation of small metal-oxo clusters in the pores after calcination may be supported by the wide peak at 702 cm^−1^ (Figure 6D). For example, this peak implies on the presence of Mn^3+^ as found in α-Mn_2_O_3_ (Mn–O stretch and O–Mn–O bend motions) (Julien et al., 2004), naturally at much lower symmetry of the Mn–O octahedral environment (only one unique Mn–O distance) (Post et al., 2020).

**FIGURE 7:**
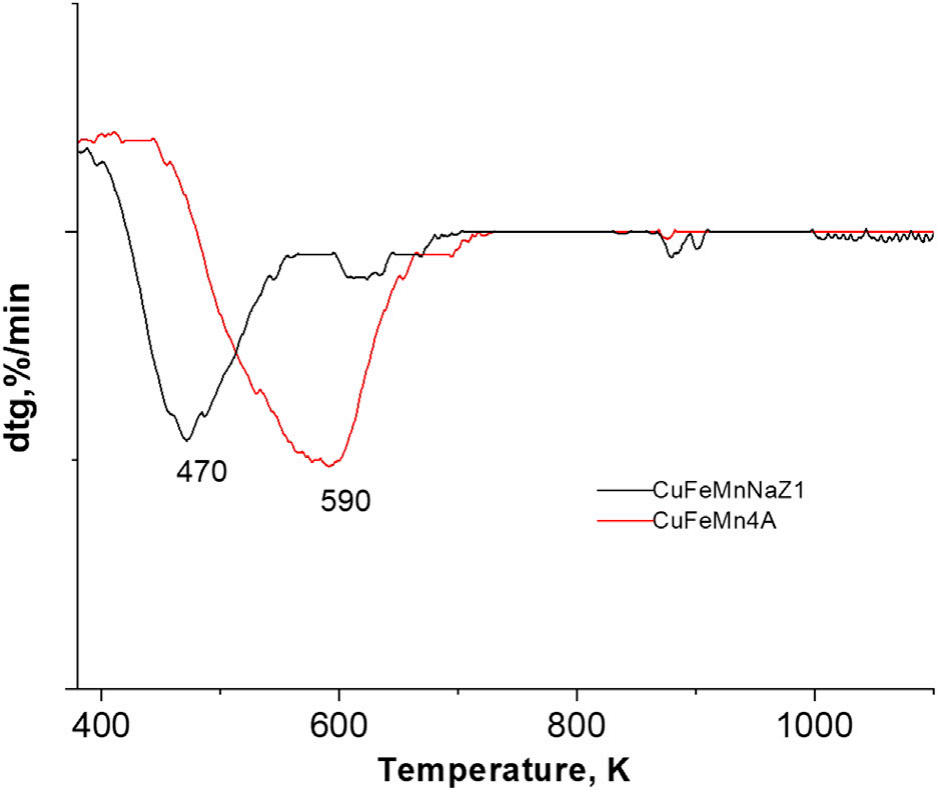
TPR profiles of the calc-CuFeMn-NaZ and calc-CuFeMn-4A samples.

TPR-TG profiles of the CuMnFe-modified materials are shown in Figure 7. TPR curves of the calc-CuFeMn-4A and calc-CuFeMN-NaZ samples show basically one reduction step.

The TPR peak, which indicates the reducibility of the materials is registered at 197°C (470 K) for calc-CuFeMn-NaZ sample, whereas in calc-CuFeMn-4A it occurred at a much higher temperature of 317°C (590 K). The differences in temperature are attributed to the formation of different types of oxo/oxide species, as well as different ratios/compositions of metal species in the two zeolite samples, as showed by Raman and TEM analyses.

In our case, the most determining factor is probably the distribution and location of metal-oxo/oxide species; in clinoptilolite-based sample the oxides are highly dispersed over the surface of zeolite and thus the sample is reducible at relatively lower temperature, while in the case of zeolite A, possible metal oxo clusters or single-metal ions, which are strongly attached to the LTA framework [e.g., Asedegbega-Nieto et al., 2010], are located in the pores, which makes the reduction more difficult.

### 3.3 Catalytic activity of thermally treated waste zeolites

For catalytic tests, we have selected metal-loaded and thermally treated zeolites calc-CuFeMn-NaZ-50 and calc-CuFeMn-4A-50. We have used toluene as a model VOC molecule as one of the most common solvents in chemical and processing industries. In all test reactions, CO_2_ and water were the only reaction products, confirming that total oxidation of toluene is the only reaction taking place. To evaluate possible synergistic effects of the three metals, as it was reported for MnNi clinoptilolite system (Ahmadi et al., 2017), we also performed the same catalytic tests for single metal modified samples of the two zeolites, i.e., calc-Cu-NaZ-n, already reported in (Logar et al., 2021), calc-Mn-NaZ-n and calc-Fe-NaZ-n (*n* = 50 mg and 150 mg) as well as calc-Cu-4A-n (Logar et al., 2021), calc-Mn-4A-n and calc-Fe-4A-n (*n* = 50 mg and 150 mg) (Supplementary Table S6).

**FIGURE 8:**
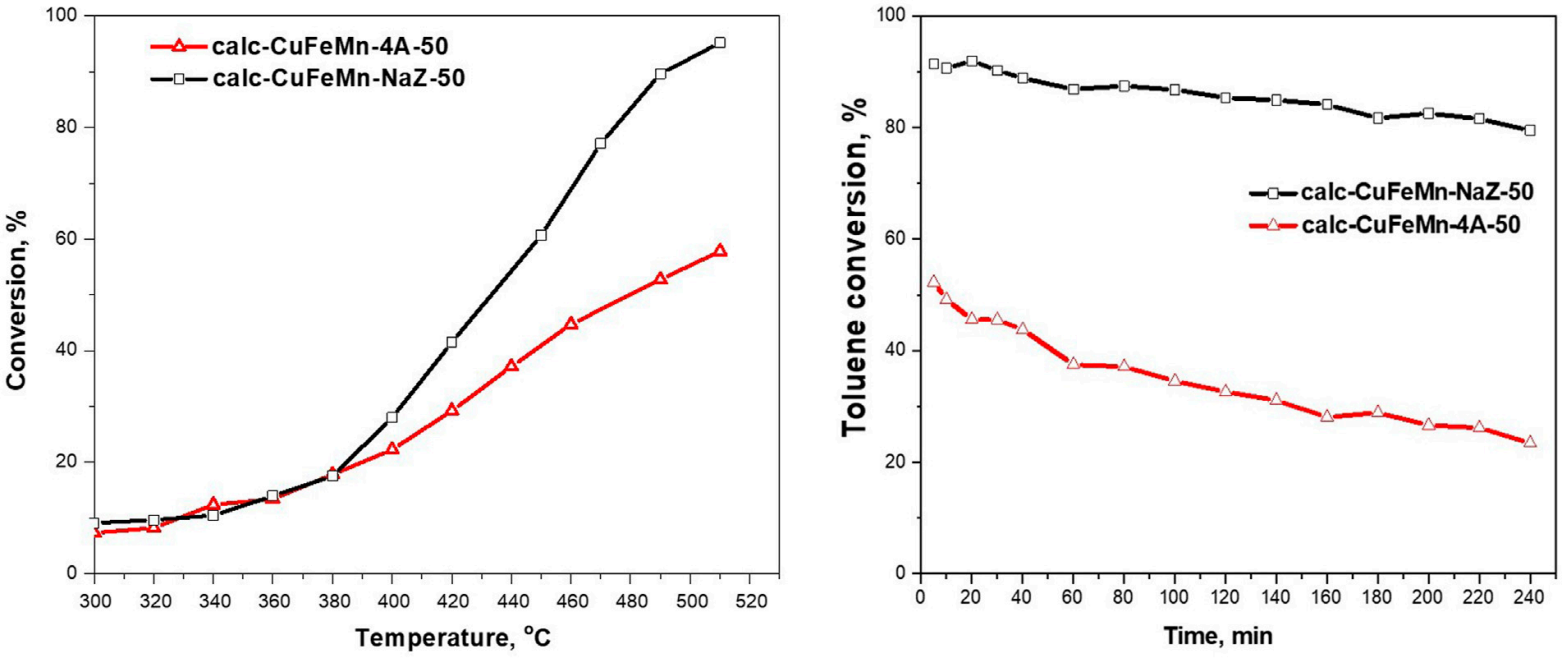
Catalytic activity of thermally treated waste zeolites as a function of temperature (left) and toluene conversion vs time on stream of two types of zeolite in catalytic reaction of total toluene oxidation (right).

Figure 8 (left) shows the activity of the two zeolite materials, calc-CuFeMn-NaZ-50 and calc-CuFeMn-4A-50, in toluene oxidation reaction. As shown in the figure, metal-loaded natural zeolite tuff proved more active (95% of toluene removed at 510 °C) than metal loaded synthetic zeolite A (up to 58% at 510°C).

The higher catalytic activity of the calc-CuFeMn-NaZ-50 catalyst could be related to the presence of finely dispersed CuO/Cu_2_O, Fe_2_O_3_ and mixed oxides (probably copper ferrite), which are detected by Raman spectroscopy and indicated by TEM, but not detectable by XRD. Furthermore, calc-CuFeMn-NaZ-50 in general possesses higher concentration of Fe (small amount of Fe is present already in zeolite tuff) than calc-CuFeMn-4A-50, which could also add to a higher concentration of active sites and higher activity. According to the Mars van Krevelen mechanism, which is widely accepted for the toluene oxidation, the higher dispersion of the metal oxide species supported on the zeolite favors the easier oxygen release which is an essential step in the catalytic process (Djinović et al., 2020; Taylor et al., 2000; Minico et al., 2000). The mechanism of oxidation suggests the adsorption of the VOC molecules to occur on the catalyst surface, their oxidation with lattice oxygen following the oxidation of the reduced catalysts with oxygen from reaction medium. The easier reduction of metal oxides in the calc-CuFeMn-NaZ-50 catalyst, proved also by TPR, leads to its higher catalytic activity in toluene oxidation. Furthermore, the surface properties (acidic and basic) of the zeolite catalyst affect the sorption of organic molecules and therefore its conversion in the oxidation reaction. Activity in total oxidation of VOC is due to the interaction of the aromatic electrons with the acidic centers of the zeolite, increasing the possibility of an electrophilic attack of adsorbed oxygen and combustion of the toluene molecules.

On the other hand, the formation of metal oxides is not confirmed for the calc-CuFeMn-4A-50 material by Raman spectroscopy and XRD. Furthermore, possible location of metal-species only in the pores of zeolite A-based samples, which are not well accessible for test toluene molecules, may add to the lower activity of calc-CuFeMn-4A-50, if compared to a favourable distribution of metal-oxo particles on the surface of clinoptilolite-based sample. As already mentioned by TPR results discussion, the type and distribution of metal strongly influence the reducibility, which occurs at a higher temperature for metal-loaded zeolite A sample (∼320 °C) and at a lower temperature in clinoptilolite-based material (∼200 °Cand that the redox properties of the formed metal oxide species in the catalysts are a crucial step in the Mars-van-Krevelen mechanism. The easier release of oxygen can be predetermined by the easier reduction of the formed metal oxides/oxo-species.

The stability test (Figure 8 (right)) revealed a small decrease in the catalytic activity for calc-CuFeMn-NaZ during the studied period, while a significant decrease in the activity of calc-CuFeMn-4A is registered. If oxide nanoparticles would be present in calc-CuFeMn-4A, we could assign this difference to the further agglomeration of the metal oxide particles on the outer zeolite A surface and/or structural degradation of zeolite framework. These are the two most common reasons for reduced activities. However, in the absence of metal oxides in calc-CuFeMn-4A and retained zeolite crystal structure, as suggested by structural analysis, the difference could be explained by further blocking of the pores and limiting access of reactant toluene molecules, caused by unfavourable migration of metal-oxo species in the pores of zeolite A/on the entrance of the pores, during stability test.

For comparison, single-metal samples were also tested. The results are presented in Supplementary Figure S3. Also here, the zeolite tuff samples perform better than synthetic zeolite A samples. Furthermore, the activity in metal-loaded natural zeolite samples is enhanced with higher amount of single metal (150 mg vs. 50 mg). The best performing Cu-loaded sample is calc-Cu-NaZ-150, for which the activity reaches 92% at 500°C [36]. The Calc-Cu-4A-50 and calc-Cu-4A-150 show similar and low catalytic activity not exceeding 20% regardless of the different copper content in the samples. Likewise, for Fe-loaded samples the best performing sample is calc-Fe-NaZ-150, which shows 97% at 500°C. Similarly, the calc-Fe-4A-50 and calc-Fe-4A-150 show similar and low catalytic activity not exceeding 20% regardless of the different iron content in the samples. The activity of Mn-loaded samples is lower regardless of the type of zeolite and concentration of metal, i.e., the highest is 72% at 500°C for calc-Mn-NaZ-50. Interestingly, the activity of calc-Mn-NaZ-150 is lower (52% at 500°C) than for calc-Mn-NaZ-50, which might be due to more pronounced pores blocking, when a higher amount of metal is loaded.

The activity of the calc-CuFeMnNaZ-50 sample is in the range of reported values for some other catalysts for the total toluene oxidation, like Cu,Fe-modified silica with interparticle mesoporosity (Djinovic et al., 2020). Some data from the literature (Ozcelik et al., 2009; Soylu et al., 2010) show also higher activities of single-metal modified clinoptilolites (Mn, Co., Fe), which reached up to 93% of toluene oxidation at 350 °C and 9.5% MnO_2_ loading and even lower temperature activity of two-metal form if compared to monometallic ones (with Mn7%Ni3%-clinoptilolite sample the toluene removal was achieved at 225°C) (Ahmadi et al., 2017). The lower activity of the calc-CuFeMn-NaZ-50 samples compared to the data from the literature is mainly due to the significantly lower concentration of metals in them than in the ones in (Ozcelik et al., 2009; Soylu et al., 2010; Ahmadi et al., 2017) (Table 2). The catalytic results obtained with other modified zeolites are summarised and presented in Supplementary Table S3. It can be clearly seen that even with 0.15 at%Cu, 0.60 at%Fe and 0.15 at%Mn in the calc-CuFeMn-NaZ-50 catalyst and lower catalyst’s amount used in the reaction we achieved almost total toluene removal at 500°C. We should mention that the needed temperature for thermal decomposition of VOCs is above 650°C.

Even though the superior catalytic activity was not achieved, the approach opened a new pathway toward an environmental problem related to the treatment of waste zeolite adsorbents. The so-prepared-catalysts’ composition could of course be further optimized with respect to their catalytic performance. In general, the suggested process includes two steps with strong environmental impact, the first one is related to the adsorption of harmful metals from polluted water and the second one is the application of waste zeolite as catalysts in removal of VOCs from polluted air. An idea for future investigations could be to select/accumulate higher content of metals from solutions thus improving the activity of the waste zeolite in VOCs removal.

## 4 Conclusion

The study confirmed the high potential of natural and synthetic zeolites for their use in wastewater treatment, i.e. rapid and efficient simultaneous removal of three metal cations from aqueous solutions was demonstrated in the systems using clinoptilolite tuff and synthetic zeolite 4A as sorbents. For both zeolite types, the sorption study showed that the selectivity of metal sorption is in the following order Fe^3+^ < Cu^2+^ < Mn^2+^ and that one type of metal cation does not exclude the immobilization of the other two metals.

The usability of thermally treated (calcined) waste metal-loaded zeolites, generated after wastewater treatment, was checked by catalytic total toluene oxidation reaction. The tests showed good performance for calcined metal-loaded clinoptilolite samples (96% conversion at 510°C) and less efficient conversion for calcined metal-loaded zeolite 4A (60% conversion at the same T). The catalytic stability test further revealed that for clinoptilolite-based materials the catalytic activity is only slightly reduced (15% in 4 h); while for zeolite 4A the catalytic activity drops to half of a starting value. The good catalytic performance of metal loaded clinoptilolite was attributed to the presence of metal-oxide nanoparticles with fine dispersion, preferably on the surface of the particles and/or in the mesopores of the clinoptilolite-based sample, while for the synthetic zeolite A sample, the lower activity and stability were attributed to the absence of accessible, well defined oxide particles and unfavourable migration of oxo-clusters in narrow pore entrances of zeolite A during catalytic reactions. In addition to higher activities, the advantage of natural zeolite support is that they are cheaper and easily available.

## Data Availability

The original contributions presented in the study are included in the article/Supplementary Material, further inquiries can be directed to the corresponding author.
