# Supplementary material for: Utilisation of waste Cu-, Mn- and Fe-loaded zeolites generated after wastewater treatment as catalysts for air treatment
Source: Front Chem. 2022 Dec 2;10:1039716. doi: 10.3389/fchem.2022.1039716 (PMC9755879; doi:10.3389/fchem.2022.1039716)
Supplement: Supplementary file 1 [file DataSheet1.PDF]

Supplementary Information
for manuscript

Utilisation of waste Cu-, Mn- and Fe-loaded zeolites generated after wastewater treatment as catalysts for air treatment

by Stankovic et al.

*** Correspondence:**
Nataša Zabukovec Logar
natasa.zabukovec@ki.si

S1. TG analysis of NaZ form of clinoptilolite tuff

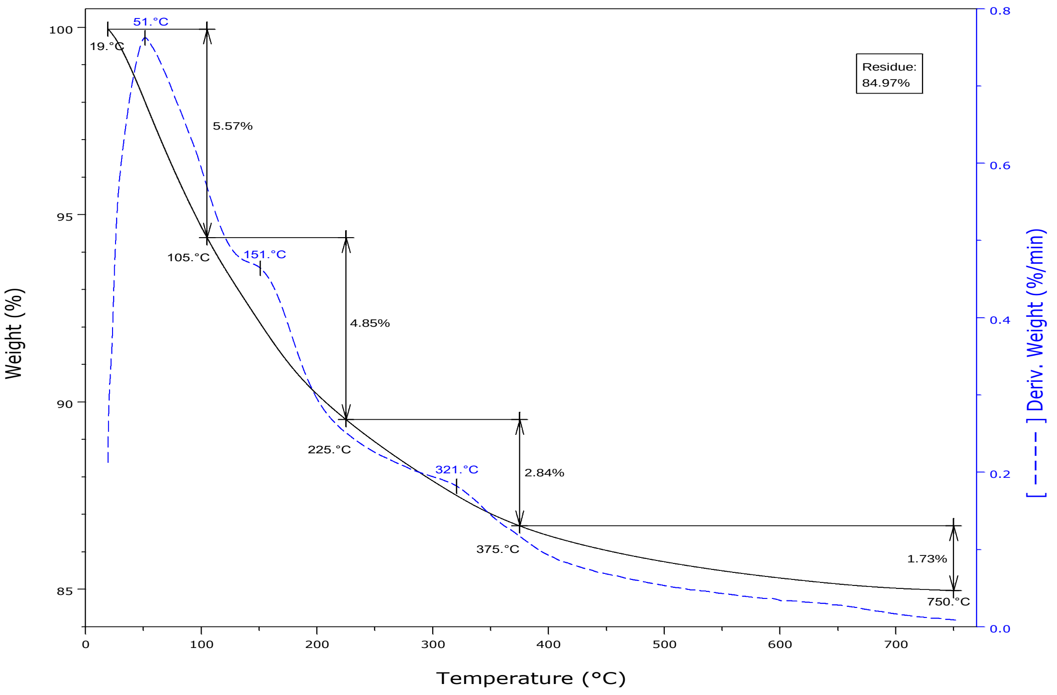

Figure S1. TG of NaZ sample.

S2. XRD patterns of single metal (Cu, Mn, Fe)-loaded zeolite tuff and synthetic zeolite A after test catalytic reaction

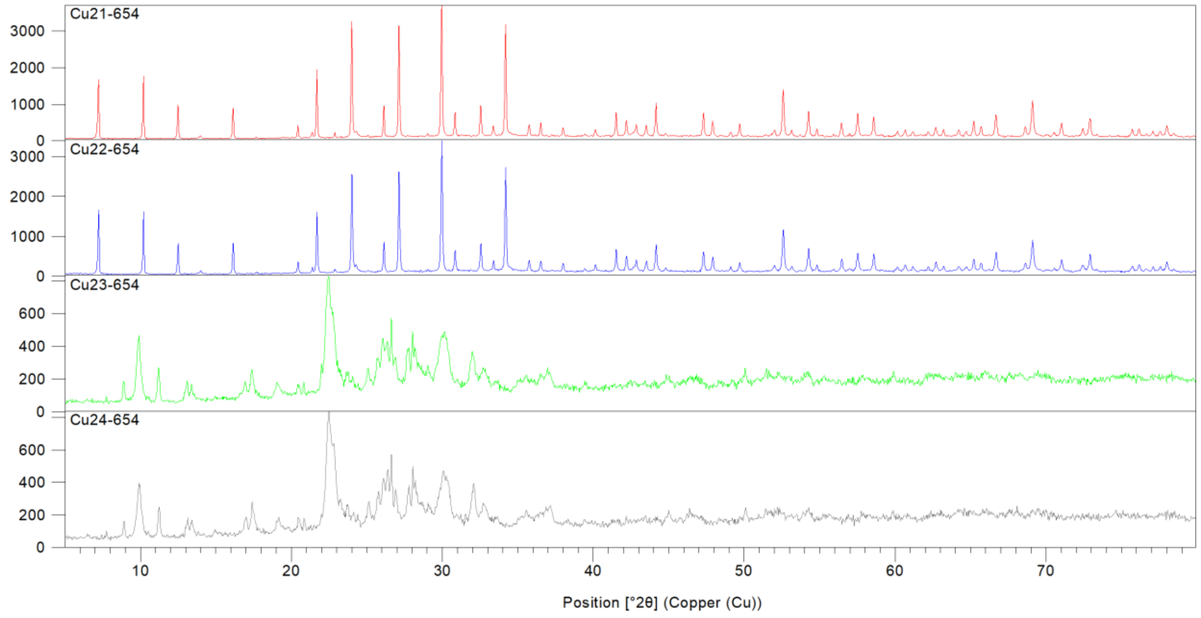

Figure S2.1. XRD patterns for (bottom to up) postcalc-Cu-NaZ-50, postcalc-Cu-NaZ-150, postcalc-Cu-A-50, and postcalc-Cu-A-150

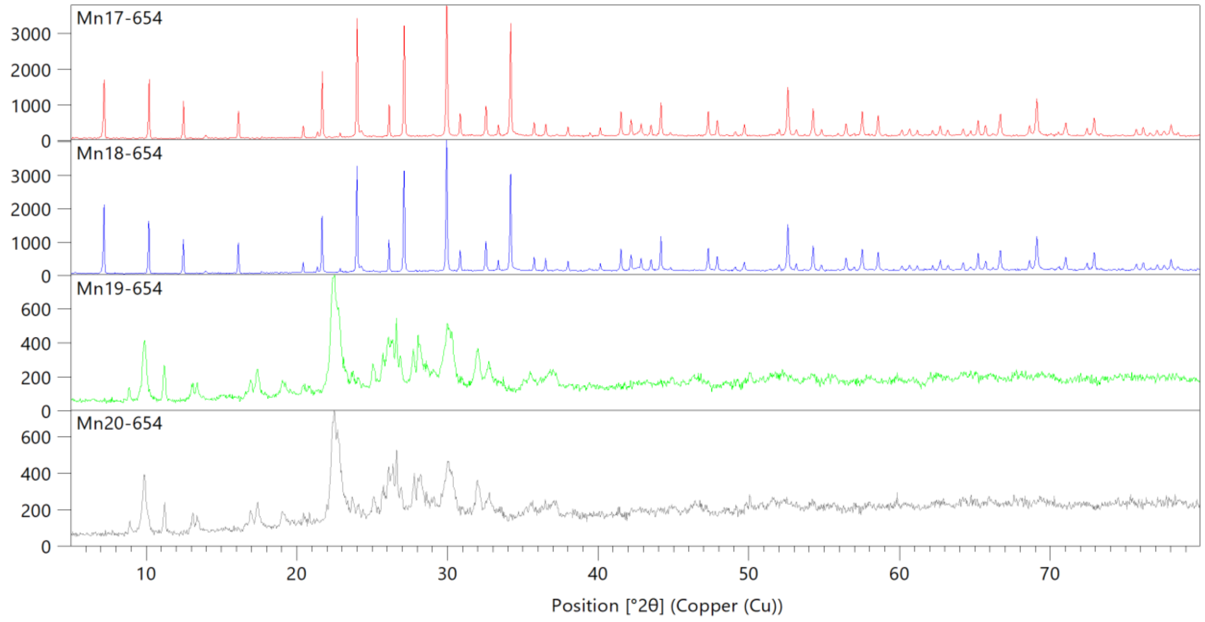

Figure S2.2. XRD patterns for (bottom to up) postcalc-Mn-NaZ-50, postcalc-Mn-NaZ-150, postcalc-Mn-A-50, and postcalc-Mn-A-150

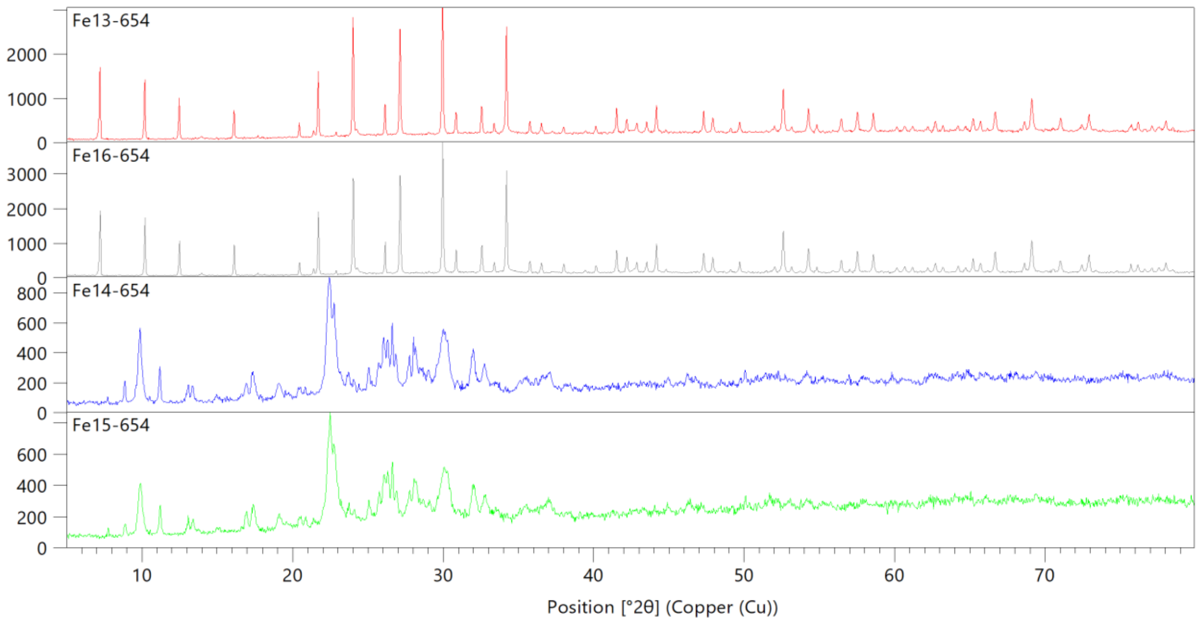

Figure S2.3. XRD patterns for (bottom to up) postcalc-Fe-NaZ-50, postcalc-Fe-NaZ-150, postcalc-Fe-A-50, and postcalc-Fe-A-150

S3. Activity of single metal (Cu, Mn, Fe)-loaded zeolite tuff synthetic zeolite A in total toluene oxidation reaction

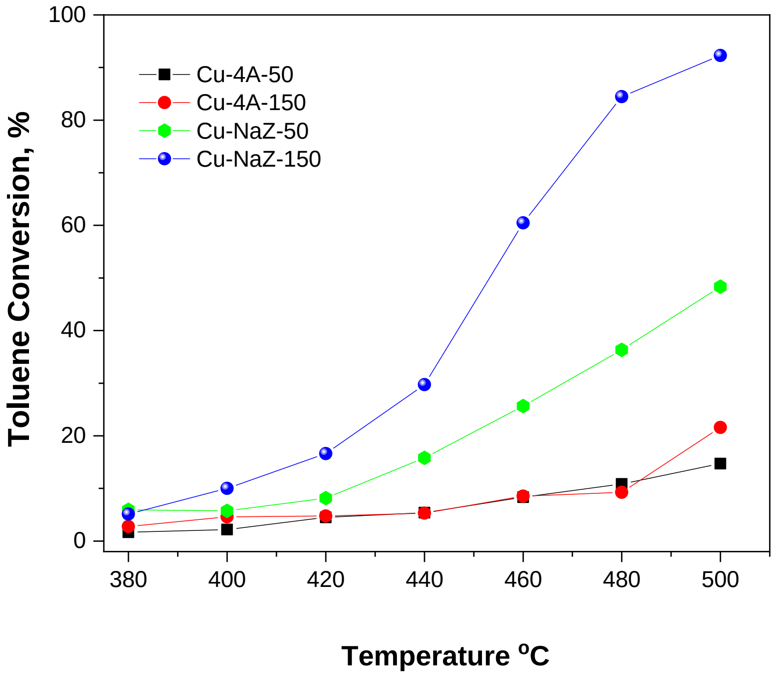

Figure S3.1. Catalytic activity of thermally treated Cu-exchanged zeolites as a function of temperature.

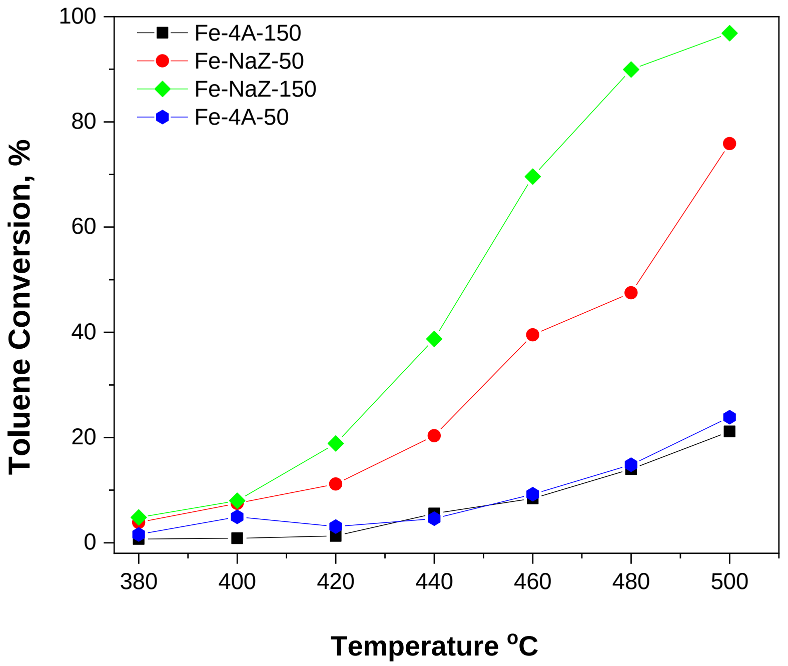

Figure S3.2. Catalytic activity of thermally treated Fe-exchanged zeolites as a function of temperature.

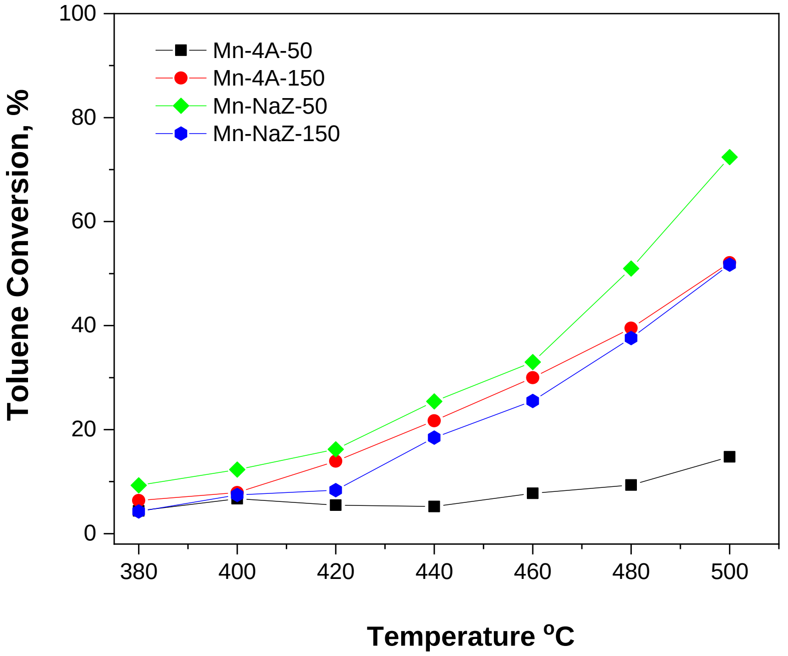

Figure S3.3. Catalytic activity of thermally treated Mn-exchanged zeolites as a function of temperature.

Table S3.1. Comparison of catalytic performance of the zeolite-based catalysts

**Catalysts**	**Reaction conditions**	**Conversion, %**	**Reference**
9.5MnO_2/Clinoptilolite	292°C, 0.15 g., GHSV = 15 000 h^{-1}, 1000 ppm toluene	100	Soylu et al., 2010
Mn7Ni3/Clinoptilolite	225°C, 0.5 g catalyst, GHSV = 10 000 h^{-1}, 3000 ppm toluene	100	Ahmadi et al., 2017
15%Cu/Clinoptilolite–CeO_2	T = 350°C, 0.5 g. Catalyst, GHSV = 6000 h^{-1}, 1000 ppm toluene	98	Yosefi et al., 2014
15%Ni/CeO_2–Clinoptilolite	T = 350°C, 0.5 g. Catalyst, GHSV = 6000 h^{-1}, 1000 ppm toluene	100	Yosefi et al., 2017
Cu(0.15at.%)Fe(0.60at.%)Mn (0.15at.%)-NaZ	T = 500°C, 0.2 g. catalyst, WHSV = 1.2 h^{-1}, 880 ppm toluene	97	This work

S4. SEM analysis

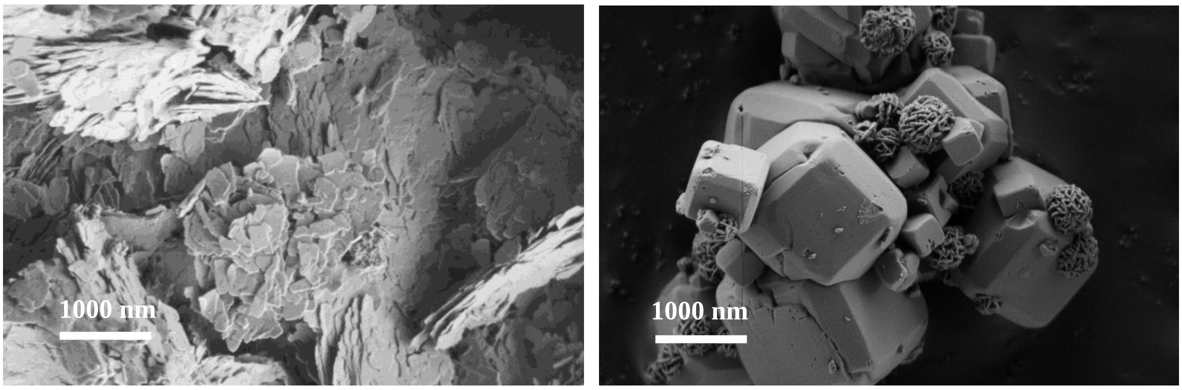

Figure S4. SEM of metal-loaded zeolite clinoptiolite tuff (left) and zeolite 4A (right)

S5. Qualitative analysis of NaZ XRD data

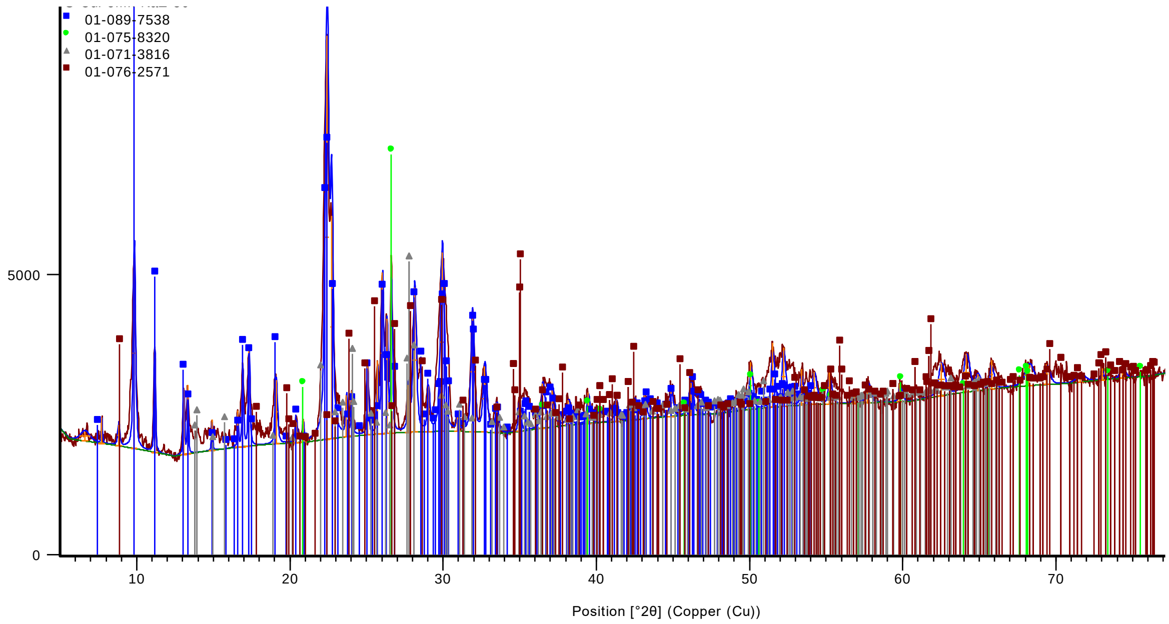

Figure 5.1. Qualitative analysis of NaZ.
PDF database reference numbers (01-089-7538-clinoptilolite, 01-075-8320-quartz, 01-071-3816-albite, 01-076-2571-muscovite)

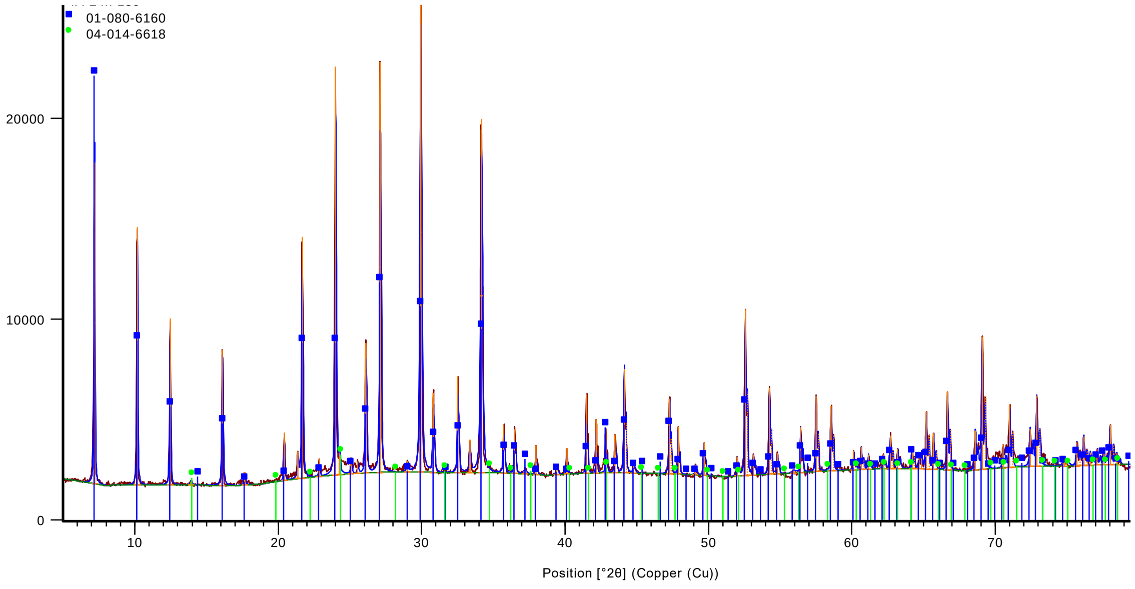

Figure 5.2. Qualitative analysis of zeolite A.
PDF database reference numbers (01-080-6160-zeolite A, 04-014-6618-sodalite)

S6. EDXS analysis

Table S6.1**.** An average elemental composition of calcined single-metal loaded samples and calcined three-metal loaded samples (after 24h of treatment) obtained by the EDX analysis (**in wt. %**). Calculated wt.% of elements from data in Table 2 (at.%) for *S-CuFeMn–NaZ-50 (24h) and S-CuFeMn–4A-50 (24h).*

**Sample**	**O**	**Na**	**Mg**	**Al**	**Si**	**K**	**Ca**	**Mn**	**Fe**	**Cu**
calc-Cu–NaZ-50 (24h)	53.68	3.42	0.54	6.64	33.56	0.67	0.37		0.48	0.64
calc-Cu–NaZ-150 (24h)	53.10	2.74	0.52	6.61	33.93	0.67	0.32		0.44	1.67
calc-Fe–NaZ-50 (24h)	54.00	3.31	0.51	6.51	33.43	0.74	0.39		1.11	
calc-Fe–NaZ-150 (24h)	53.87	2.97	0.45	6.32	33.35	0.67	0.38		1.99	
calc-Mn–NaZ-50 (24h)	53.73	3.45	0.50	6.52	33.67	0.69	0.35	0.50	0.58	
calc-Mn–NaZ-150 (24h)	53.62	2.71	0.42	6.68	33.58	0.63	0.38	1.49	0.49	
calc-Cu–4A-50 (24h)	51.42	13.29		16.77	17.79					0.73
calc-Cu–4A-150 (24h)	51.79	12.49		16.37	17.58					1.77
calc-Fe–4A-50 (24h)	51.10	13.44		16.93	17.99				0.54	
calc-Fe–4A-150 (24h)	51.41	11.87		16.32	18.53				1.87	
calc-Mn–4A-50 (24h)	51.70	13.01		16.81	17.90			0.58		
calc-Mn–4A-150 (24h)	50.67	12.63		16.89	18.16			1.65		
calc-CuFeMn–NaZ-50 (24h)	53.47	2.74	0.48	6.61	33.63	0.66	0.38	0.47	1.03	0.53
calc-CuFeMn–4A-50 (24h)	51.23	12.37		16.83	17.69			0.62	0.64	0.62
S-CuFeMn–NaZ-50 (24h) – adopted from Table 2	53.68	2.86	0.39	6.21	33.30	0.76	0.26	0.41	1.65	0.48
S-CuFeMn–4A-50 (24h) – adopted from Table 2	50.01	12.44		17.23	18.55			0.52	0.67	0.67

S7. TEM data

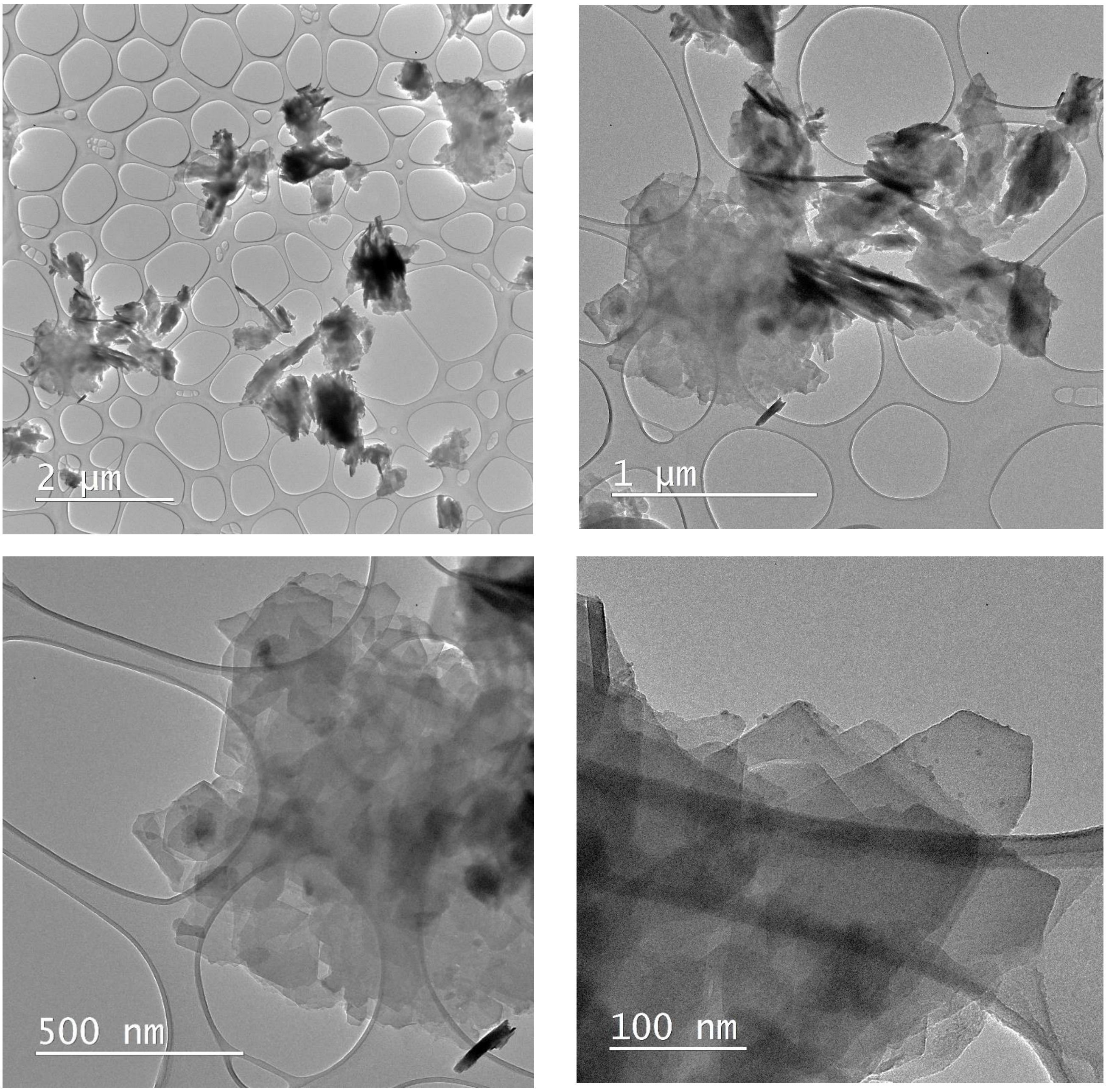

Figure S7.1: TEM micrographs of CuFeMn-NaZ-50 sample at lower magnifications

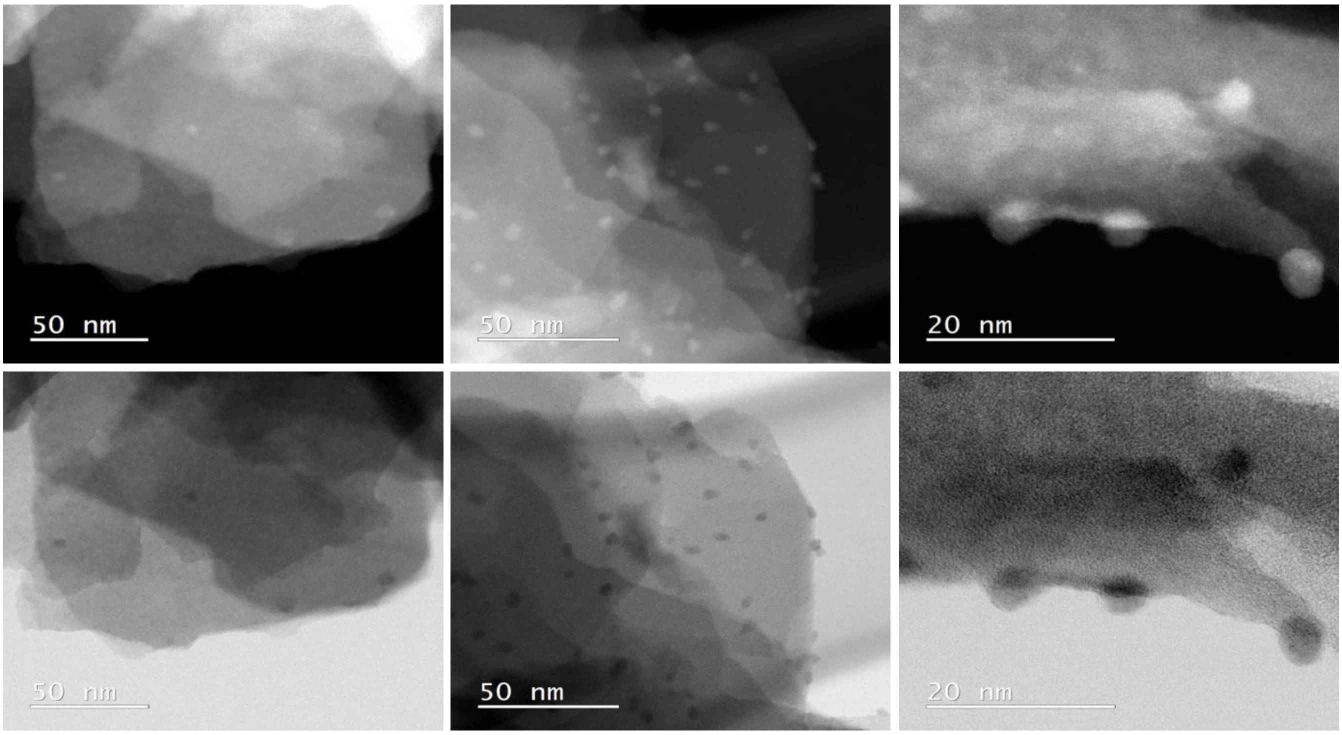

Figure S7.2: HAADF-STEM (upper) and BF-STEM (lower) images of CuFeMn-NaZ-50 sample

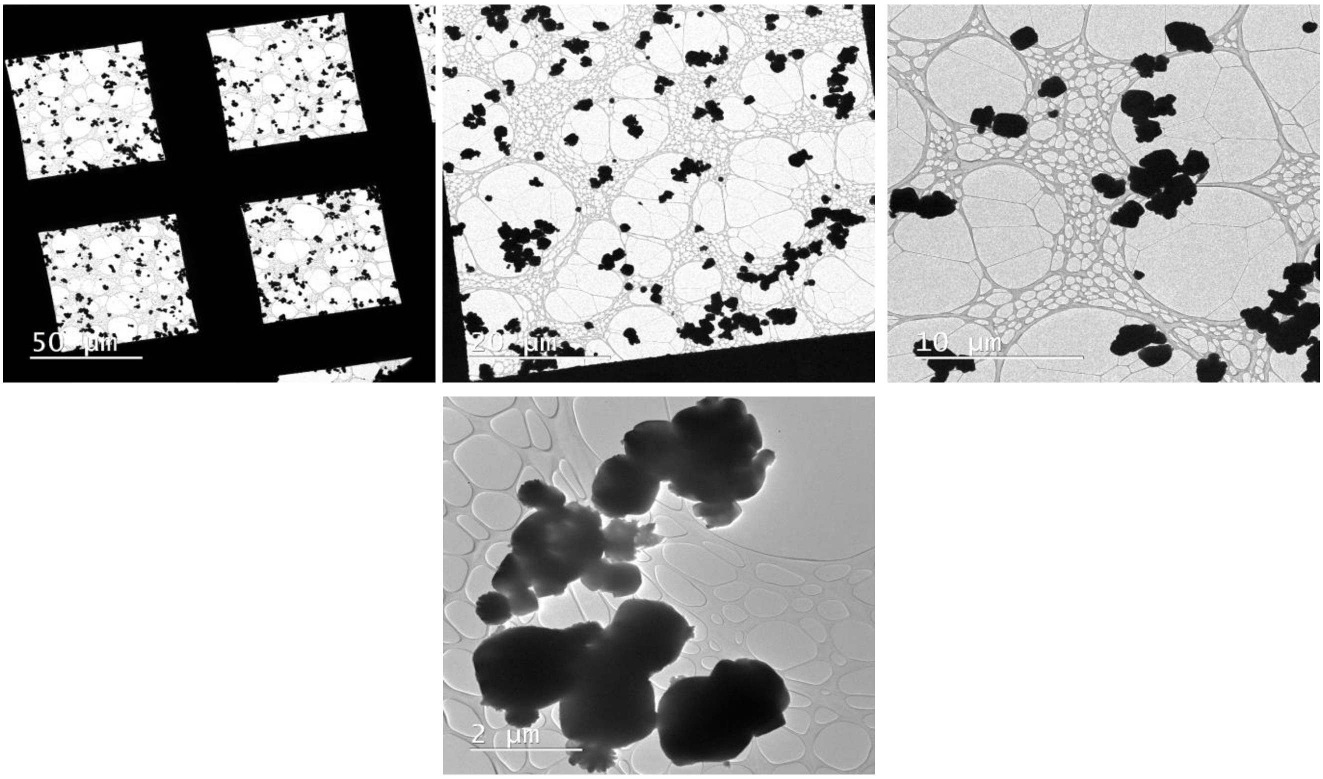

Figure S7.3: TEM micrographs of of CuFeMn-4A-50 sample at lower magnifications

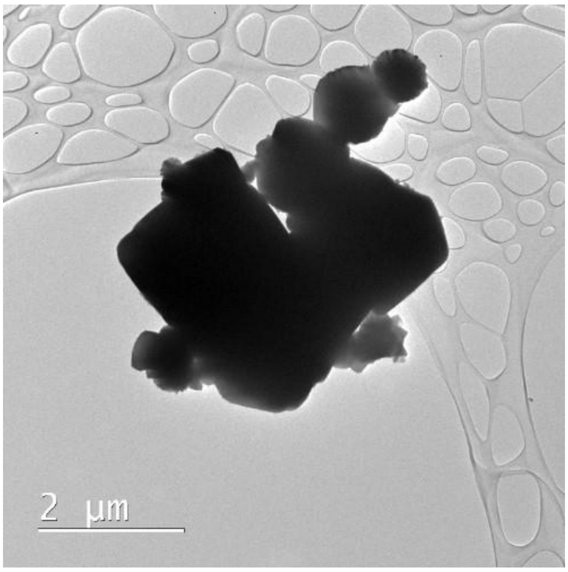

Figure S7.3: TEM image of calc-CuFeMn–4A-50 sample.

S8. FTIR, Raman and UV/vis data

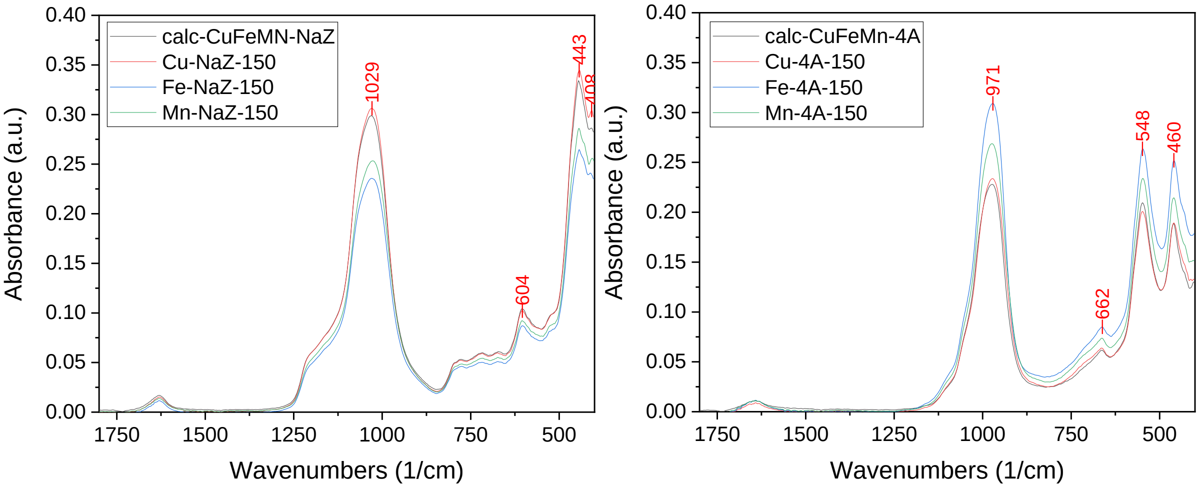

(a) (b)

Figure S8.1: ATR-FTIR spectra of samples. In (a) series with natural zeolite is shown while facet (b) depicts series of spectra with synthetic zeolite 4A.

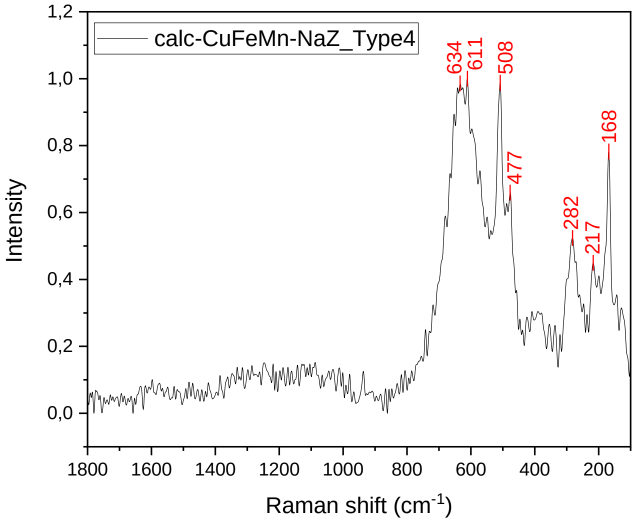

Figure S8.2: Raman spectrum of sample calc-CuFeMn-NaZ-50 showing the fourth type of spectra.

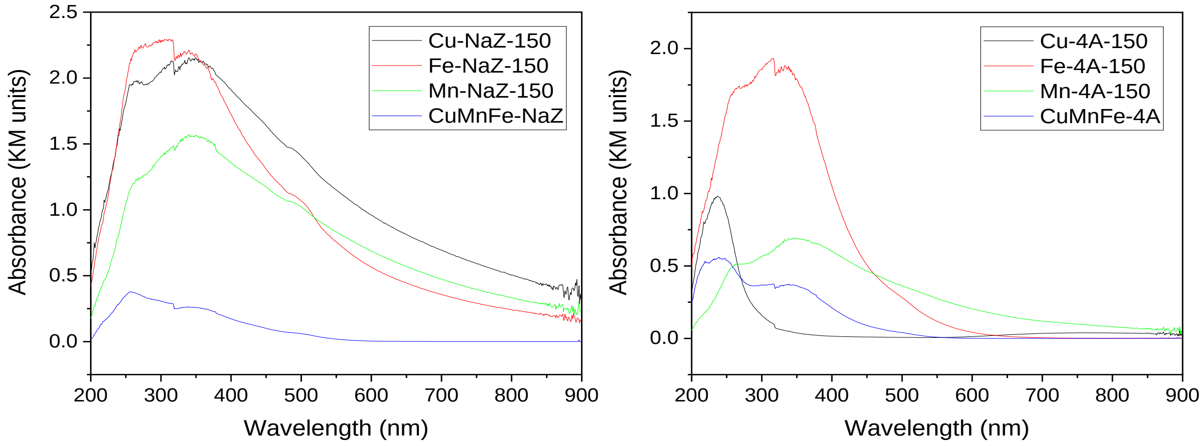

(a) (b)

Figure S8.3: UV/vis DRS spectra of samples. In (a) series with natural zeolite is shown while facet (b) depicts series of spectra with synthetic zeolite 4A.

S9. Ar and CO_2 sorption measurements

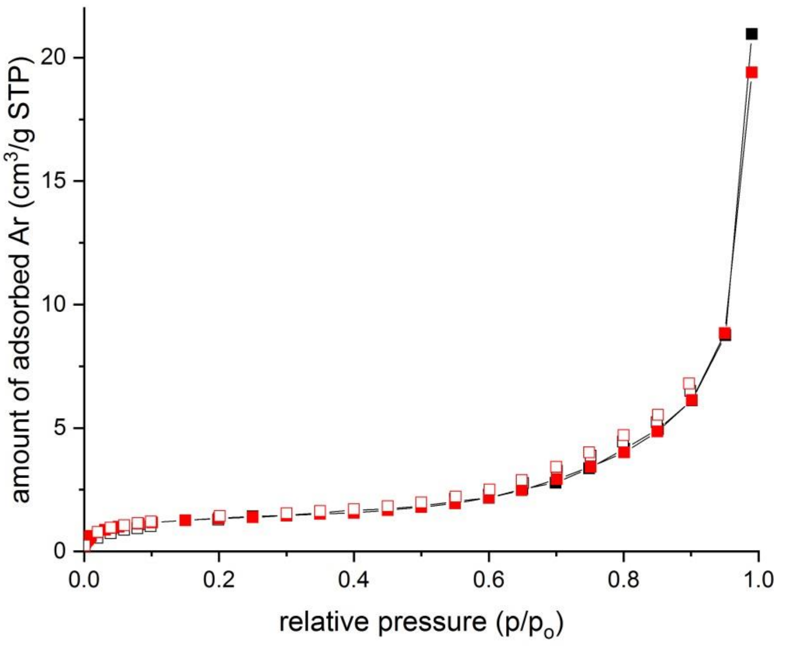

Figure S9.1: Ar isotherms of CuFeMn-4A-50 samples measured at 87 K. Black plots show as-synthesized material (S-CuFeMn–4A-50) whereas red plots calcined sample (calc-CuFeMn-4A-50). Full symbols – adsorption, empty symbols – desorption.

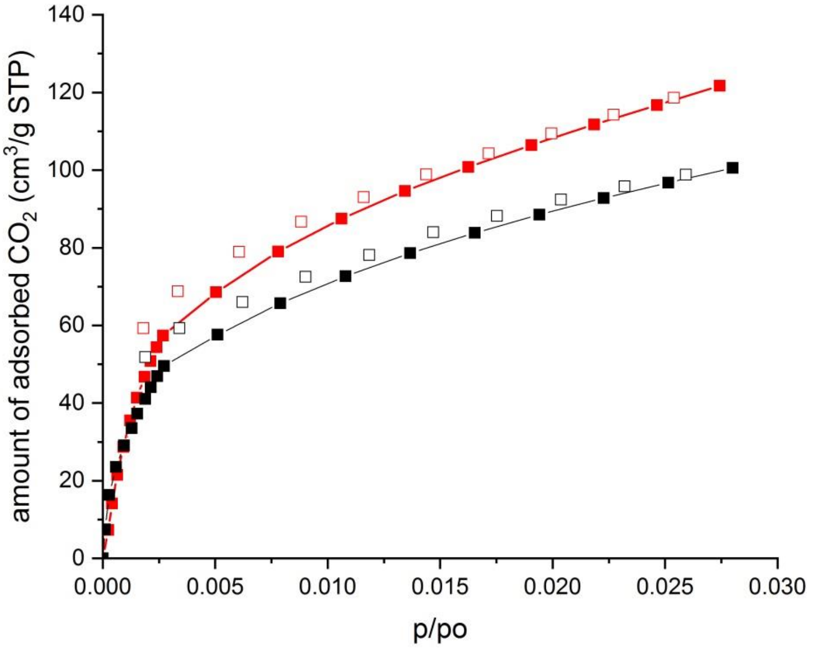

Figure S9.2: CO_2 isotherms of CuFeMn-4A-50 samples measured at 273 K. Black plots show as-synthesized material (S-CuFeMn–4A-50) whereas red plots calcined sample (calc-CuFeMn-4A-50). Full symbols – adsorption, empty symbols – desorption.

Table S9.1: Comparison of BET specific surface areas of CuFeMn-4A-50 samples based on Ar and CO_2 isothermal data.

Sample	[1]S_{BET-Ar} (m^2 g^{-1})	[2]$S_{BET-CO2}$ (m^2 g^{-1})
S-CuFeMn–4A-50	3.5	381
calc-CuFEMn-4A-50	3.6	411

[1]BET surface area calculated from argon isotherms measured at 87 K. [21]BET surface area calculated from carbon dioxide isotherms measured at 273 K.

S10. Single-metal sorption performance

Table S10.1. Comparison of sorption performance of selected zeolite-based sorbents

**Material**	**Concentration of metals in starting solution (mg L^{-1})**	**pH**	**Time (h)**	**T (°C)**	**Metal removal from solution, % of initial concentration**	**Reference**
Clinoptilolite NaZ	100-400(Cu)	/	24	25-55	NA	Stojakovic et al., 2011
Clinoptilolite NaZ	50-800(Cu)	4.5 -5	24	25	NA	Milicevic et al., 2022
Clinoptilolite NaZ	50-400(Mn)	3-8	24	25-65	NA	Rajic et al., 2017
Zeolite X	2500-50000(Cu)	6-7	24	25	NA	Mokrzycki et al., 2022
Clinoptilolite NaZ	63,5(Cu)	5-6	48	25	96	Logar et al., 2021
Zeolite 4A	100(Cu)	7-9	48	25	99	Logar et al., 2021
Clinoptilolite NaZ	50(Cu), 50(Mn), 50(Fe)	5-6	24	25	72(Cu), 70(Mn), 78(Fe)	This work
Zeolite 4A	50(Cu), 50(Mn), 50(Fe)	7-9	24	25	84(Cu), 78(Mn), 86(Fe)	This work
